# A Model of Brain Circulation and Metabolism: NIRS Signal Changes during Physiological Challenges

**DOI:** 10.1371/journal.pcbi.1000212

**Published:** 2008-11-07

**Authors:** Murad Banaji, Alfred Mallet, Clare E. Elwell, Peter Nicholls, Chris E. Cooper

**Affiliations:** 1 Department of Biological Sciences, University of Essex, Colchester, United Kingdom; 2 Department of Medical Physics and Bioengineering, University College London, London, United Kingdom; Indiana University, United States of America

## Abstract

We construct a model of brain circulation and energy metabolism. The model is designed to explain experimental data and predict the response of the circulation and metabolism to a variety of stimuli, in particular, changes in arterial blood pressure, CO_2_ levels, O_2_ levels, and functional activation. Significant model outputs are predictions about blood flow, metabolic rate, and quantities measurable noninvasively using near-infrared spectroscopy (NIRS), including cerebral blood volume and oxygenation and the redox state of the Cu_A_ centre in cytochrome *c* oxidase. These quantities are now frequently measured in clinical settings; however the relationship between the measurements and the underlying physiological events is in general complex. We anticipate that the model will play an important role in helping to understand the NIRS signals, in particular, the cytochrome signal, which has been hard to interpret. A range of model simulations are presented, and model outputs are compared to published data obtained from both *in vivo* and *in vitro* settings. The comparisons are encouraging, showing that the model is able to reproduce observed behaviour in response to various stimuli.

## Author Summary

Monitoring the brain noninvasively is key to solving various biological and clinical problems. Near-infrared spectroscopy (NIRS) is a technique that can measure changes in the colour of the brain. The brain has an absolute requirement for oxygen; the spectroscopically observed colour changes are due to the proteins that deliver (haemoglobin) and consume (mitochondrial cytochrome *c* oxidase) oxygen. Haemoglobin changes colour when it binds oxygen. The changes in cytochrome *c* oxidase are due to the electron occupancy (reduction) of a particular copper metal centre in the enzyme. The way that the state of this enzyme changes in various situations is poorly understood. Currently there is no theoretical model that can be used to decode simultaneously all of the spectroscopic changes in these proteins, and thus limited information about the underlying biochemistry and physiology can be extracted from the NIRS signals. We therefore constructed such a model, ensuring that it is consistent with the scientific literature, *in vivo* data, and the underlying thermodynamic principles. The model was able to predict the physiological and spectroscopic responses to a wide range of stimuli, including changes in brain activity and oxygen delivery. It is likely to be of significant value to a wide range of clinical and life science users.

## Introduction

In recent years there has been widespread use of near infrared spectroscopy (NIRS) to monitor brain oxygenation, haemodynamics and metabolism [1],[2]. Initially the primary chromophores of interest were oxy- and deoxy-haemoglobin, with changes (termed ΔHbO2 and ΔHHb, respectively) being measured using differential spectroscopy systems [3]–[5]. Technical developments made possible the measurement of absolute tissue oxygen saturation (TOS). This quantity has been variously labelled rSO2 (regional saturation of oxygen, Somanetics INVOS systems), TOI (tissue oxygenation index, Hamamatsu NIRO systems) and StO_2_ (tissue oxygen saturation, Hutchinson InSpectra systems). TOS provides a percentage measure of mean oxygen saturation across all vascular compartments in the tissue of interest. TOS has been used extensively as a marker of tissue oxygenation in a range of applications [6]–[9] but its relationship to underlying physiology is still under investigation [10],[11].

In addition to the haemoglobin chromophores, the Cu_A_ centre in cytochrome *c* oxidase (CCO) is a significant NIR absorber. Measurement of the changes in oxidation level of this centre give rise to a signal, here referred to as the ΔoxCCO signal, which has been extensively investigated as a marker of cellular oxygen metabolism [12]–[15]. A number of clinical studies have been performed to elucidate its role as a measure of cerebral well being [16]–[18].

Although in the case of TOS and ΔoxCCO there are no obvious “gold standard” measurements against which a direct experimental validation can be performed, these NIRS signals undoubtedly encode information of biological and, potentially, clinical importance on tissue oxygen levels, blood flow, metabolic rate (CMRO_2_), and other underlying state variables in the brain. However the mapping between NIRS signals and the underlying variables is not straightforward, as a number of different causes may give rise to the same signal changes. The data on CCO redox state is particularly difficult to interpret because of the potential complexity of the correlations between physiological changes and mitochondrial redox states [12],[19].

Thus in order to correctly interpret and maximise the clinical usefulness of the information that can be extracted from NIRS data, a model of the underlying physiology is required. This is our aim in this paper. The model we construct is based on thermodynamic principles, and is to date the only model which attempts to predict the state of the Cu_A_ centre in cytochrome *c* oxidase in an *in vivo* setting. It is designed to be able to simulate responses to physiologically and clinically important stimuli (listed below), and is able to reproduce several experimental data sets including both *in vivo* data, for example on NIRS signal changes during functional activation [5], and *in vitro* data on mitochondrial flux and redox state during hypoxia and uncoupling [20]. Moreover our simulations suggest important practical conclusions: For example, that the ΔoxCCO signal contains information independent of that contained in the other NIRS signals, and that physiological variability between individuals has the potential to affect its relationship with the haemoglobin signals.

The model is designed to respond to four input stimuli, which have been chosen both because they are physiologically important, and because there is considerable data on the response of NIRS signals to these inputs. The stimuli can be expected to cause changes in the different NIRS signals via a variety of different physiological pathways. They are

- Blood pressure changes (e.g., [8],[21])
- Changes in arterial O_2_ levels (e.g., [22])
- Changes in arterial CO_2_ levels (e.g., [23])
- Functional activation (e.g., [5],[24])

One key consideration has been to keep the model small enough to allow eventual optimising of key parameters to an individual's data. This would be required if the signals were to be used to interpret physiological changes in an individual, for example in the clinical setting. For this reason rather than attempting to append a model of mitochondrial metabolism to the large and complex BRAINCIRC model [25], we have used this model as the basis for a much simpler model.

In order to increase readability, model differential equations, and tables of model variables and parameters are presented in Text S1. The model was written and simulated in the open source BRAINCIRC interface [26] and is available for download [27], complete with instructions on how to reproduce each of the simulation plots presented in this paper.

## Methods

### Model Structure

The model consists essentially of two components. The first is a submodel of the cerebral circulation, which is known to respond in complicated ways to a variety of stimuli – physical, chemical and neuronal [28]. Though much of the physiology is still under investigation, there are a variety of more or less simplified models which attempt to capture some features of this control. Among these are the models of Ursino and co-workers ([29],[30] for example), the model of Aubert and Costalat [31], and the BRAINCIRC model [25] described in [32] and still under active development. All of these models have contributed to the construction of the model described in this paper.

The second component of the model presented here is a submodel of mitochondrial metabolism. Several such models exist, notably the models of Korzeniewski and co-workers (e.g., [33]) and Beard and coworkers [34],[35]. These models have also played a large part in the construction of our model, and processes here are often either caricatures or refinements of processes in these models. The two components of the model are linked via oxygen transport and consumption.

**Figure 1:**
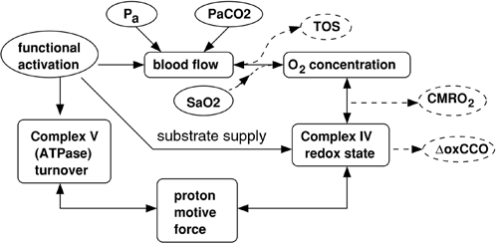
Summary of the main inputs, variables and processes in the model. Model inputs are enclosed in solid ovals, while outputs are enclosed in dashed ovals. *P_a_* is arterial blood pressure, SaO2 is arterial oxygen saturation level, PaCO2 is arterial CO_2_ level. TOS and ΔoxCCO are NIRS signals defined in the text.

The basic structure of the model is illustrated in Figure 1. In order to aid model validation, a smaller mitochondrial model appropriate to *in vitro* situations will also be introduced later. In particular this model omits all processes relating to blood flow, with oxygen being supplied directly to the mitochondria.

### Compartments

Following the normal simplification in most chemical models, all chemical reactions are assumed to take place in solution in compartments. A reference brain volume is assumed (although never needed explicitly) and other volumes are calculated as fractions of this reference volume. Thus “blood volume” and “mitochondrial volume” will refer to blood/mitochondrial volume *per unit brain volume*. Processes take place at two sites: in a blood compartment, divided into an arterial compartment with variable volume, a capillary compartment with negligible volume, and a venous compartment with fixed volume; and a mitochondrial compartment with fractional volume Vol_mit_ which can be interpreted as ml mitochondrial volume per ml tissue. The arterial volume Vol_art_ and venous volume Vol_ven_ are expressed as fractions of *normal* total blood volume, so that in normal conditions, Vol_art_+Vol_ven_ = 1. In other words they measure ml arterial/venous blood per ml normal blood volume.

### pH Buffering

Following [33], the presence of buffers in the mitochondria serves effectively to enlarge mitochondrial volume as seen by protons. We define an effective mitochondrial volume for protons Vol_Hi_ = R_Hi_Vol_mit_ where
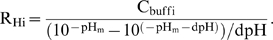
C_buffi_ and dpH are constants.

### Units

As discussed above, all volumes are taken as fractions of a reference volume and are thus, strictly speaking, dimensionless. When the reference volume is not clear the complete units will be presented. In general, chemical concentrations are millimolar (mM), with the reference volume being implicit (so for example concentration of a substance Y in mitochondria has units millimoles Y per litre of mitochondrial internal volume). The exceptions are when a unit conversion is carried out to follow convention or to facilitate comparison with data, as in the case of NIRS quantities which are generally in *μ*M and where the reference volume is brain volume even when the quantity is confined to some specific compartment. All blood pressures and partial pressures of gases are in mmHg. For readability, units will be generally omitted from the text but are presented in the Sections B and C of Text S1.

### Blood Flow Regulation

The first component of the model is a basic representation of the mechanics of cerebral blood flow. This part of the model is a simplification of the detailed biophysics in [29], where regulation occurs at two sites—a proximal and a distal arterial compartment, each responding to stimuli differently. We constructed a version of this model with a single compartment which was able to reproduce steady state responses to stimuli adequately, and so in our model here, a single compartment is used. Certain processes are omitted, including the viscous response of blood vessels, and the complexities of the venous circulation. The conductance of the circulation, *G*, determines cerebral blood flow CBF according to the ohmic equation
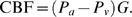

*P_a_* and *P_v_* are arterial and venous blood pressure respectively, which are parameters external to the model. Cerebral blood flow (CBF) in the model means the volume of blood which flows through a unit volume of tissue in unit time. *G* is taken to be a function of a typical “radius” *r* of the resistance vessels according to the Poiseuille law:
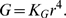

*K_G_* is simply a constant of proportionality. *r* is determined by the balance of forces
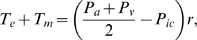
(1)where *T_e_* and *T_m_* are, respectively, the elastic and muscular forces developed in the vessel wall, both functions of the radius, and *P_ic_* is extravascular pressure (assumed to be constant). (*P_a_*+*P_v_*)/2 is an average intravascular pressure. Following [29] the elastic tension is given an exponential dependence on radius:
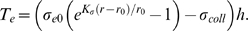
(2)Here *σ_e_*_0_, *K_σ_*, *r*_0_ and *σ_coll_* are parameters, while *h* is the vessel wall thickness, set by conservation of wall volume according to the equation:
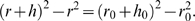
(3)
*h*_0_ represents wall thickness when vessel radius is *r*_0_.

The muscular tension is given by
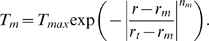
(4)
*T_m_* has a bell-shaped dependence on radius, taking value *T_max_* at some optimum radius *r_m_*. *r_t_* and *n_m_* are parameters determining the shape of the curve. Maximum muscular tension *T_max_* is a crucial quantity, and is affected by all stimuli which cause changes in vascular smooth muscle tension. To this end it is useful to define a dimensionless quantity *μ* which represents the level of regulatory input, giving
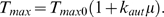

*T_max_*_0_ is a constant and *k_aut_* is a control parameter, normally set to 1, but which can be lowered to simulate loss of a vessels ability to respond actively to stimuli. *μ* varies between a minimum value of *μ_min_* and a maximum value of *μ_max_*. The level of regulatory input depends on the level of stimuli capable of producing a response in vascular smooth muscle. These stimuli are combined into a dimensionless quantity *η* which determines *μ* via a sigmoidal function:
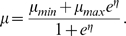
A single compartment with these functional responses was found in preliminary simulations to be able to reproduce experimentally observed steady state responses well. Further details are presented in the results and in Text S1.

In the model, four quantities are capable of producing direct or indirect responses in vascular smooth muscle and hence affecting *η*: arterial blood pressure, oxygen levels (taken for simplicity to be mitochondrial oxygen levels), arterial CO_2_ pressure PaCO2, and demand, which we represent as a dimensionless parameter *u*. In its action within mitochondria, *u* may be identified with the ADP/ATP ratio, while in its effect on blood flow it can be seen as the level of the substrates connected with neurovascular coupling. *u* is introduced in order primarily to simulate, via a single parameter, the events occurring during functional activation. In order to construct *η*, we define four quantities 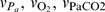 and *v_u_*. These are essentially *P_a_*, [O_2_], PaCO2 and *u*, respectively, passed through first order filters, in order to represent possibly different time constants associated with each of these stimuli:
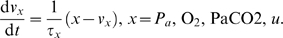
(5)The time constants *τ_x_* control how long it takes for each stimulus to have a vasoactive effect. Given that blood flow regulation in response to a single stimulus often involves multiple processes occurring on different time scales (for example direct and metabolic effects of hypoxia), use of a single time constant for each stimulus is necessarily an approximation. *η* is chosen to be linear in all stimuli:
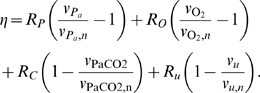
The parameters *R_P_*, *R_O_*, *R_C_* and *R_u_* represent the sensitivities to changes in the different stimuli while *v_x,n_* represents the normal value of *v_x_*, so that at normal values of all stimuli *η* = 0, and hence *μ* = (*μ_min_*+*μ_max_*)/2.

Collapsing the complexity of the biology into a single quantity *η* will necessarily have some pitfalls. However for our purposes here, the simple form of *η* is sufficient.

### Oxygen Transport and Consumption

Knowledge of oxygen levels in blood is necessary both in order to interpret haemoglobin related NIRS signals, and also in order to calculate oxygen transport to tissue. It is conceptually simplifying to consider oxygen binding sites on haemoglobin as the chemical of interest, with concentration four times the concentration of haemoglobin. Thus oxyhaemoglobin concentration will refer to the concentration of filled oxygen binding sites on haemoglobin.

Arterial oxyhaemoglobin concentration [HbO_2,a_] is calculated from arterial saturation SaO2 and total haemoglobin concentration in arterial and venous blood [Hbtot] (assumed constant) via [HbO_2,a_] = SaO2[Hbtot].

A quantity *J*_O2_ can be defined as the rate of oxygen flux from blood to tissue (in micromoles O_2_ per ml tissue per second). A key requirement is that total O_2_ supplied to the tissue is matched by oxygen delivery. This requirement is encoded in an equation
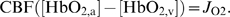
(6)[HbO_2,a_] and [HbO_2,v_] are the arterial and venous concentrations of oxygenated Hb respectively. From the venous oxyhaemoglobin level we can calculate a venous saturation SvO2 = [HbO_2,v_]/[Hbtot].

The concentration of oxyhaemoglobin will clearly vary along the capillary bed. Defining a typical capillary oxygen saturation ScO2 = (SaO2+SvO2)/2 we can use this to calculate a typical capillary oxygen concentration
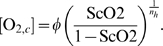
(7)
*φ* is the concentration of dissolved oxygen giving half maximal saturation, while *n_h_* is the Hill exponent of the dissociation curve. Clearly choosing this form for dissolved oxygen ignores possible complications arising from the Bohr effect (see [36] for example).

By choosing a simplified form for the level of capillary oxygen, we run the risk of miscalculating oxygen delivery. An example of a more complete treatment using a distributed model can be found in [37]. In order to investigate the possible errors introduced by this simplification a distributed model was solved numerically and the true average capillary oxygen concentration compared to that calculated from Equation 7. The results are presented in Section D of Text S1. The approximation causes consistent overestimation of capillary oxygen concentration introducing an error of approximately 2.5 percent in normal circumstances. During severe ischaemia this error can grow to 6 percent. In order to minimise model complexity, we accept this level of error in the current model.

The process by which oxygen is supplied to the mitochondria is assumed to be diffusive occurring at a rate
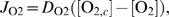
(8)where [O_2_] is the mitochondrial oxygen concentration, and *D*_O2_ is the diffusion coefficient. In order to ensure that arterial oxygen supply can never exceed tissue oxygen delivery (and thus avoid venous oxygenation becoming negative) we do not allow the value of supply to exceed CBF[HbO_2,a_], i.e. we set *J*_O2_ = min{*D*_O2_([O_2,*c*_]−[O_2_]),CBF[HbO_2,a_]}. More details on this crude methodology for modelling a process which properly requires PDE modelling are given in Appendix C of [32]. For *in vivo* simulations where oxygen saturation may decrease significantly, in order to avoid non-smooth behaviour we use the smooth approximation to the function 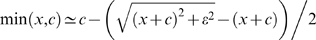, choosing *ε* in this case to be CBF_n_[HbO_2,a,n_]/10.

Equations 6–8 collectively serve to determine the values of [HbO_2,v_], [HbO_2,c_], [O_2,c_] and *J*_O2_ and need to be solved simultaneously.

### Arterio-Venous Volumes and NIRS Measures of Blood Oxygenation

A key variable measurable using NIRS is tissue oxygen saturation (TOS), the average saturation level of blood in the brain for which an absolute value can be obtained. This can be expressed as a value between 0 and 1 or as a percentage, and in the equations below we choose the former. In addition, changes in tissue oxy-, deoxy-, and total haemoglobin concentration (as distinguished from blood concentrations), termed ΔHbt, ΔHbO2 and ΔHHb respectively and measured in *μ*mol(l tissue)^−1^ can be calculated.

In order to calculate TOS, we need only the relative volumes Vol_art_ and Vol_ven_ (and no value for the fractional volume of blood per unit brain volume). Ignoring the capillaries, which are assumed to have small volume, we get
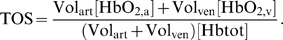
Next we assume that Vol_art_ is proportional to *r* ^2^ so that 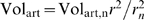 where Vol_art,n_ and *r_n_* are the normal values of Vol_art_ and *r*. Dividing the expression for TOS through by the normal arterial volume, Vol_art,n_, and defining normal arterio-venous volume ratio AVRn = Vol_art,n_/Vol_ven_, then gives:
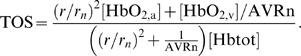
In order to define the other NIRS quantities we require some estimate of absolute blood volume in the tissue. So we define a parameter Vol_blood,n_ in (ml blood)(ml tissue)^−1^, and get the tissue concentrations of total, oxy- and deoxy-haemoglobin in *μ*mol(l tissue)^−1^ as, respectively:
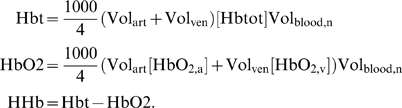
The factor of 1000 arises from conversion from mM to *μ*M, while division by 4 occurs because of our definition of Hb as *binding sites* on haemoglobin. Multiplication by Vol_blood,n_ is to convert to tissue concentrations. NIRS signals ΔHbt, ΔHbO2 and ΔHHb are then
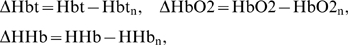
where Hbt_n_, HbO2_n_ and HHb_n_ are normal values of Hbt, HbO2 and HHb.

### Basic Mitochondrial Submodel Structure

The second key component of the model is a basic submodel of mitochondrial dynamics centred in particular on the oxidation state of the Cu_A_ centre in cytochrome *c* oxidase. The inspiration for this model comes from the detailed models of [33] and [34], and the abstract model in [38]. However, in order to minimise model size, many of the processes in [33] and/or [34] have been omitted: in particular phosphate and ADP/ATP transport, and the adenylate kinase and creatine kinase reactions. Further, the behaviour of complexes I-III has been lumped into a single process. On the other hand somewhat more detail has been included in the treatment of complex IV (cytochrome *c* oxidase) with a view to more accurate information on the redox state of the Cu_A_ centre. It is worth mentioning that the simplifying assumption of a single site of oxidative metabolism ignores the diverse roles of neurons and astrocytes in brain energy metabolism.

**Figure 2:**
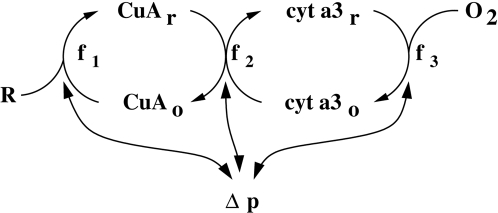
Schematic representation of the mitochondrial submodel. The Cu_A_ centre is reduced by some reducing substrate, termed R. It in turn passes its electrons on to a terminal substrate, cyta_3_. Finally cyta_3_ is oxidised by oxygen. All processes can in general produce proton motive force Δp, by pumping protons out of the mitochondrial matrix. As a result, they are also inhibited by Δp. The rates of the three processes – initial reduction of Cu_A_, electron transfer to cyta_3_ and final oxidation of cyta_3_, are termed *f*_1_, *f*_2_ and *f*_3_, respectively.

Two redox centres in cytochrome *c* oxidase are identified explicitly, Cu_A_, and the terminal electron acceptor cytochrome a_3_ (henceforth termed cyta_3_). Each of these centres can exist in either an oxidised or a reduced form. A reducing substrate transfers electrons (directly or indirectly) to Cu_A_, which in turn transfers its electrons to cyta_3_. Finally cyta_3_ transfers its electrons to oxygen. These three electron transfers, which we will refer to as reaction 1, reaction 2 and reaction 3, occur at rates *f*_1_, *f*_2_ and *f*_3_. These rates are taken to be the rates of transfer of **four** electrons between substrates. They are accompanied by the pumping of protons across the mitochondrial membrane, and hence both create and are affected by the proton motive force Δp (also termed PMF, discussed below). The structure of this submodel is shown in Figure 2.

From here on, we represent the concentration of oxidised and reduced Cu_A_ by CuA_o_ and CuA_r_ respectively. Similarly oxidised and reduced cyta_3_ are represented by a3_o_ and a3_r_ respectively. The total concentrations of Cu_A_ and cyta_3_ in mitochondria are assumed constant at some value cytox_tot_.

The proton motive force Δp has both a chemical and an electrical component and has the form
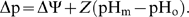
Here ΔΨ is the mitochondrial inner membrane potential, pH_o_ is pH in the intermembrane space assumed to be a constant or controllable parameter. *Z* = *RT*/*F* where *F* is the Faraday constant, *R* is the ideal gas constant, and *T* is the absolute temperature. The dynamics of ΔΨ are discussed below.

Protons move across the mitochondrial membrane in both directions. A quantity *p*_1_ of protons are pumped out during the reduction of four Cu_A_ centres, and *p*_2_ are pumped out during their oxidation, and *p*_3_ are pumped during the final oxidation of cyta_3_. *p_tot_* = *p*_1_+*p*_2_+*p*_3_ is thus the total number of protons pumped out of the mitochondria during the reduction of one molecule of O_2_. The value of *p*_1_, and hence *p_tot_*, will depend on the reducing substrate.

### Proton Entry into the Mitochondrial Matrix

The protons pumped out of mitochondria during electron transfer return into the mitochondria via leak channels at rate *L_lk_*, and via processes associated with ATP production (i.e. through Complex *V*, and during ADP/ATP and phosphate translocation) at a rate *L_CV_*. Thus the total return of protons into the mitochondria occurs at rate *L* = *L_CV_*+*L_lk_*. Following [33] the leak rate is exponentially dependent on Δp:
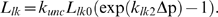

*L_lk_*_0_ and *k_lk_*_2_ are parameters controlling the sensitivity of the leak current to changes in Δp. *k_unc_* is a control parameter, normally set to 1, used to simulate the effect of adding uncouplers to the system. It is only altered during simulations of the simplified model of isolated mitochondria described below.

*L_CV_* depends on both Δp and the demand *u*. The form
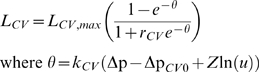
is chosen. If we identify the demand parameter *u* with an (appropriately rescaled) ADP/ATP ratio, we see that this form is similar to that for the rate of complex *V* in [33]. It is also qualitatively similar to the form in [39] despite the apparent complexity of the form in that reference. The parameter Δp*_CV_*_0_ is the value of Δp at which, given normal demand, *L_CV_* goes to zero. *k_CV_* controls the sensitivity of the rate to changes in Δp. *r_CV_* controls the relative sizes of maximal and minimal rates of *L_CV_*. If *n_A_* protons enter the matrix for every molecule of ADP phosphorylated, the actual rate of ADP phosphorylation is *L_CV_*/*n_A_*. The current consensus value of *n_A_* is given as 4.33 in [40]. Note that because of differences in the constructions of the two models, the parameter *n_A_* has a somewhat different meaning to its counterpart in [33].

Following the methodology in [35],[41], the rate of change of ΔΨ depends only on the flows of protons across the membrane and is given by
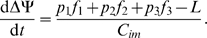

*C_im_* is the capacitance of the mitochondrial inner membrane.

### Electron Transfer and Proton Pumping

We now return to reactions 1, 2 and 3 with rates *f*_1_, *f*_2_ and *f*_3_. For simplicity each of these rates refers to the transfer of four electrons. The processes associated with rates *f*_1_ and *f*_2_ are assumed to be reversible. Assuming first order kinetics for *f*_1_ gives
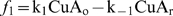
where k_1_ and k_−1_ are the forward and backward rate constants for the reaction. Although the details of how the rate constants change with changes in Δp are not known in advance, the equilibrium for the reaction can be set from energetic principles: Associated with *f*_1_ we have a free energy
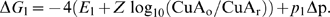
The important quantity *E*_1_ is discussed further in Section C of Text S1. Setting Δ*G*_1_ = 0 determines the equilibrium constant of the reaction Keq_1_, giving
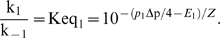
To allow for inhibition by changes in the proton motive force, k_1_ is set as
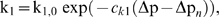
where k_1,0_ is the value of k_1_ at normal Δp. Since demand or experimental set-up may influence the redox state of the initial reducing substrate k_1,0_ is not a constant (details in Section C of Text S1). The exponential term reflects inhibition of the forward rate by Δp, and the strength of this inhibition is controlled by the parameter *c_k_*_1_. The backward rate constant is then determined from the equilibrium constant:
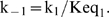



A very similar process can be used to set *f*_2_. Again, forward and backward rate constants k_2_ and k_−2_ are assumed, giving
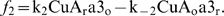
This time the free energy is
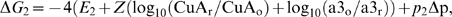
giving the equilibrium constant Keq_2_

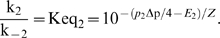
k_2_ is then set as
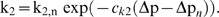

*c_k_*_2_ controls the effect of changes in Δp on k_2_. The backward rate constant is simply
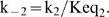
Reaction 3 is assumed to be irreversible, and its rate *f*_3_ is set as
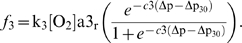
(9)


The quantities *c*3 and Δp_30_ are parameters controlling the sensitivity of *f*_3_ to Δp. From the above form it is possible to calculate an apparent second-order rate constant for the reaction taking place at zero PMF as
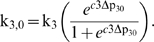
(10)Values of this parameter can be experimentally measured [42] and the measured values are used to determine the value of k_3_ in the model.

As *f*_3_ is the rate of oxygen consumption it is used to calculate the crucial model output:
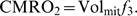
(11)In order to simplify the model we have assumed that control of cytochrome *c* oxidase is via Δp alone, ignoring the fact that changing ΔpH and ΔΨ can have different effects on cytochrome *c* oxidase turnover [43].

### Redox State of Cu_A_: The ΔoxCCO Signal

The NIRS ΔoxCCO signal can be identified as the change, in *μ*M, in the tissue concentration of oxidised Cu_A_. In order to model this quantity, we define
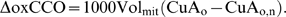
The factor of 1000 is to convert from mM to *μ*M, while multiplication by Vol_mit_—mitochondrial volume as a fraction of tissue volume—converts from mitochondrial to tissue concentration.

### A Simplified Mitochondrial Model

**Figure 3:**
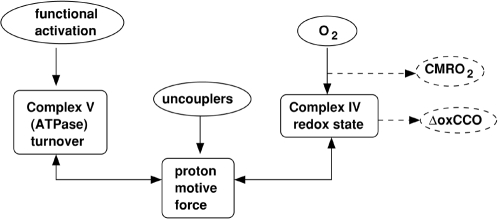
Summary of the main variables and processes in the simplified model. As in Figure 1, inputs are enclosed in solid ovals, while outputs are enclosed in dashed ovals. Components connected with blood flow have been removed from the model. O_2_ levels are now directly settable.

Apart from the model described above, in order to set parameters and compare model behaviour to experimental data a simpler submodel is also constructed. This model will be referred to as *the simplified model* while the model described above will be referred to as *the full model*. The simplified model is designed to simulate *in vitro* experiments on mitochondrial solutions, and so omits a number of processes in the full model. A schematic of this model is shown in Figure 3.

The key differences between the simplified mitochondrial model and the full model are that all processes and feedback involving blood flow are removed. Mitochondrial O_2_ becomes a control parameter rather than a model output, and the reducing substrate is not automatically assumed to be NADH, but may be chosen to be other substrates such as succinate or TMPD. The simplified model can also model experimental data involving uncouplers: These are molecules, generally protonophores, that uncouple oxygen consumption from oxidative phosphorylation, allowing rapid electron transfer with no ATP synthesis. Data from experiments such as that in [20] can then be used for model parameter setting or model validation.

## Results/Discussion

We intend our model to be able to reproduce standard, well-understood experimental phenomena; however, we also wish to use it to gain insight into areas where the physiology and biochemistry underlying the changes in the ΔoxCCO signal are poorly understood, especially quantitatively. To this end we have explored the behaviour of the model under a range of conditions.

### Autoregulation Curves and Steady State Behaviour

**Figure 4:**
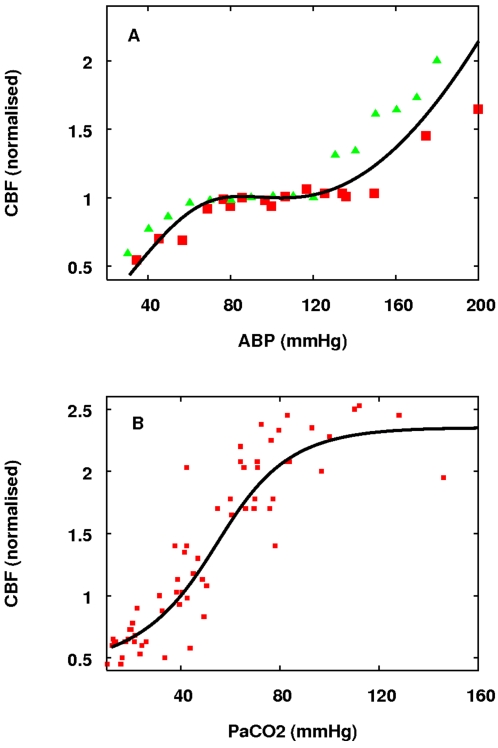
The response of model steady state CBF to blood pressure and PaCO2 changes. (A) Response to arterial blood pressure changes with data from [44] (red squares) and [45] (green triangles) for comparison. (B) Response to PaCO2 changes with data from [48] (with normal resting blood flow taken as 40 ml/min/100 g) for comparison.

The steady state response of cerebral blood flow to changes in blood pressure gives rise to “autoregulation” curves with blood flow being insensitive to changes in blood pressure around the physiological value [44]–[47]. This is obviously key behaviour that our model must be able to reproduce. Steady state responses of cerebral blood flow to other stimuli, in particular PaCO2, are also well characterised experimentally [48]. The model steady state blood flow responses to changes in blood pressure and CO_2_ levels are plotted in Figure 4.

The pressure autoregulation curve is consistent with experimental curves (e.g. the autoregulation curve in [44] constructed from data in [46],[47]) and modelled curves (e.g. using the model in [29]). Data from these studies was used to set model parameters as described in Section E of Text S1. The value of *R_C_* has been set so that model steady state response to changes in PaCO2 is consistent with published data [48]. Data from a hypercapnia study described below suggests that the magnitude of this response may vary between individuals.

### Behaviour of the Model during Functional Activation

Functional activation provides a repeatable challenge giving rise to discrete changes in metabolic demand, which can be assumed to be primarily cerebral. Since its inception in 1993 [49]–[52], the study of functional activation by NIRS (fNIRS) has rapidly become one of the main drivers in the development of NIR technology for monitoring the human brain. Yet there have been few studies focusing on the ΔoxCCO signal, despite its potential to inform on the critical question of neurovascular coupling. In 1999, a paper reported on oxidation of ΔoxCCO during fNIRS [15]. Despite a number of attempts to dismiss this result as an optical artefact, the basic finding has resisted such explanations [53]. However, whether the oxidation can be explained physiologically (effect of increased oxygen delivery) or biochemically (effect of increased ATP turnover) is not clear.

**Figure 5:**
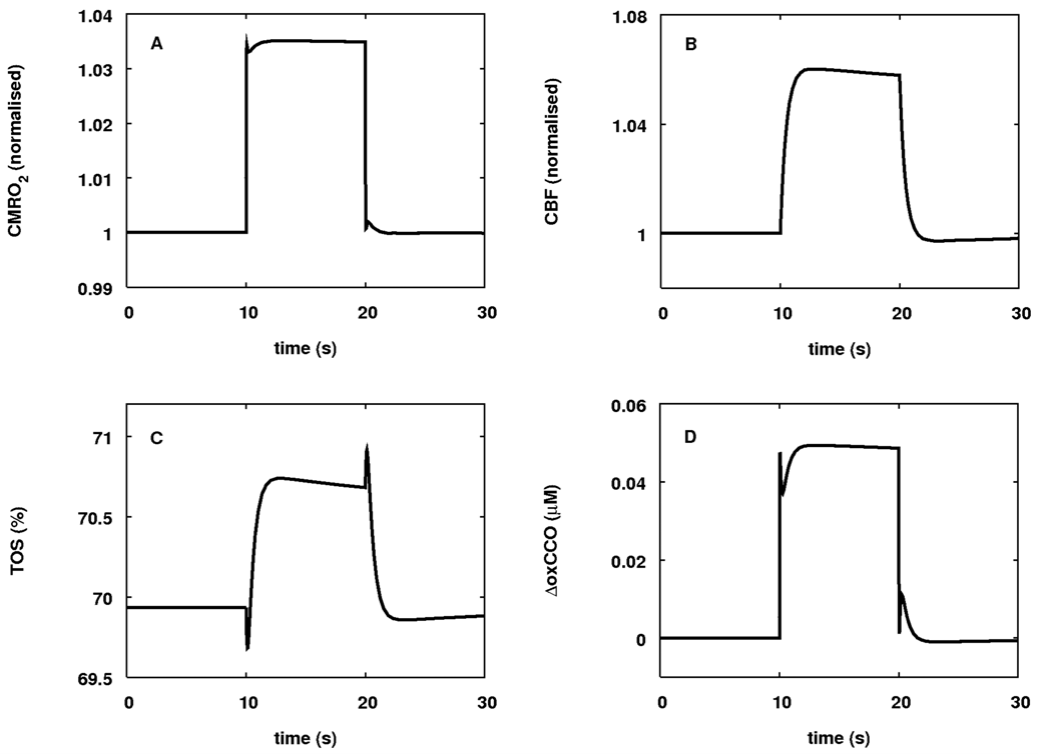
Model responses to a step up in demand. (A) Change in CMRO_2_ (normalised). (B) Change in CBF (normalised). (C) Change in TOS (percent). (D) Change in ΔoxCCO (*μ*M). All parameters are held at normal values apart from *u* which is stepped up from 1 to 1.2 for a ten second duration, giving rise to an approximately 3.5 percent increase in CMRO_2_ and an approximately 6 percent increase in blood flow. TOS increased by a little under 1 percent, and ΔoxCCO also increased by about 0.05 *μ*M corresponding to an oxidation of just under 1 percent.

In order to shed light on such questions, functional activation was simulated in the model, via a step up in the demand parameter *u*. A ten second activation was simulated by running the model at normal parameter values for 10 seconds, followed by a 10 second increase in *u*, followed by a further ten seconds at baseline. The responses of various quantities are plotted in Figure 5.

As expected, the increase in blood flow more than compensates for the increase in CMRO_2_ so that TOS goes up. The ratio of changes in blood flow to changes in CMRO_2_ is consistent with the data in [54] where a ratio of 2∶1 is typical, although higher values are reported in [55]. Also clear from the data is that at normal parameter values an increase in demand causes oxidation of Cu_A_, and hence an increase in the ΔoxCCO signal consistent in direction, but smaller in magnitude (by about 50 percent) than the typical traces in [5]. Below we show that, perhaps surprisingly, this effect is not primarily dependent on an increase in blood flow and blood oxygenation.

**Figure 6:**
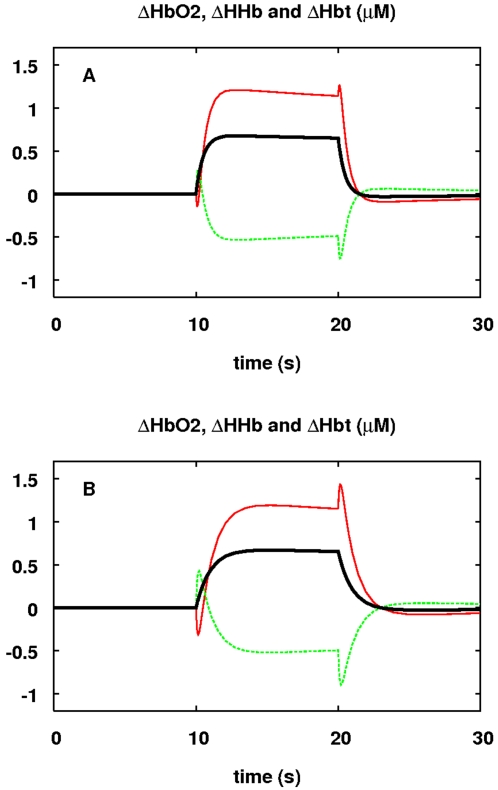
Response of haemoglobin signals to a step up in demand. The response in *μ*M of ΔHbO2 (red), ΔHHb (green) and ΔHbt (black) to a step up in demand. The stimulus and parameter values are as in Figure 5. In (A) *τ_u_* = 0.5 s (the default value). In (B) *τ_u_* = 1 s. With the slower response time, there is more pronounced transient behaviour including a clear initial decrease in ΔHbO2 before it starts to increase.

The behaviour of the other NIRS signals—ΔHbO2, ΔHHb and ΔHbt—during functional activation is plotted in Figure 6. Changing the time constant associated with demand (*τ_u_*) affects the shape of the response, and the magnitude of a slight initial increase in deoxygenated haemoglobin before it starts to drop.

Both the levels and direction of change of the haemoglobin signals are comparable with previous experimental data [24], although the magnitudes predicted are somewhat higher than reported in [5].

Consistent with the analysis in [38], both the size and the *direction* of ΔoxCCO change in response to functional activation are sensitive to a number of model parameters including the baseline PMF and values of the standard redox potentials. One interesting question is whether the effect is driven solely by the increase in cerebral blood flow associated with functional activation. A simple way to test this is by abolishing the response of blood flow to demand by setting *R_u_* = 0. This reduces the ΔoxCCO increase (by about 40 percent) but does not abolish it (results not shown).

**Figure 7:**
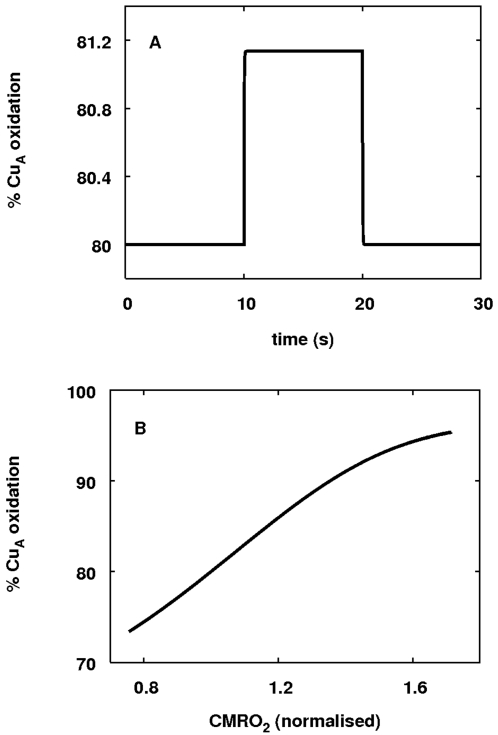
Response of Cu_A_ redox state in the simplified model to changes in *u*. (A) The time course of oxidised Cu_A_ in response to functional activation. As in the *in vivo* simulations, *u* was changed from 1 to 1.2 for a ten second duration, resulting in an approximately 1 percent increase in Cu_A_ oxidation. (B) The steady state level of Cu_A_ oxidation in response to varying levels of activation. *u* was varied from 0.2 to 100 resulting in variation in CMRO_2_ from 80 to 170 percent of baseline. Cu_A_ oxidation increased steadily.

In this light it is interesting to run an analogous simulation involving a step up in demand on the simplified mitochondrial model. Such a change can be identified with a transient increase in the ADP/ATP ratio in an *in vitro* situation. As in the *in vivo* case, there was a small but significant oxidation of Cu_A_. To see whether this oxidation is a robust response to activation, the level of activation was varied so that CMRO_2_ varied between 80 percent and 170 percent of baseline. The results of both simulations are plotted in Figure 7.

As is clear from Figure 7, increased demand oxidises Cu_A_ even in the simplified model where there is no change in oxygen level. Qualitatively similar results are obtained when an increase in demand is replaced with uncoupling. These results suggest the important conclusion that the change in the ΔoxCCO signal during functional activation is primarily associated with changes in proton motive force rather than being slaved to changes in oxygen levels. The ΔoxCCO signal thus appears to encode information about cerebral metabolic state independent of that contained in the other NIRS signals.

**Figure 8:**
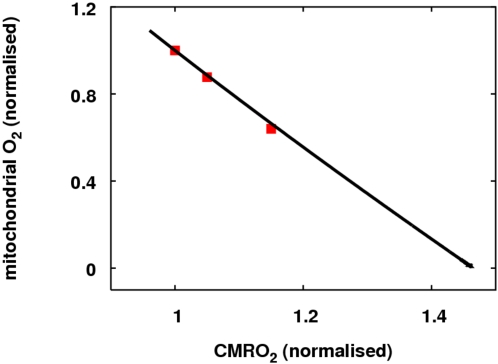
Relationship between CMRO_2_ and mitochondrial oxygen levels during activation. The full model was run with parameter *R_u_* set to zero so that an increase in demand had no effect on blood flow. Increasing *u* allowed increases in CMRO_2_ up to approximately 145 percent of baseline. The three data points shown are calculated from Figure 2 of [55] in which predictions on how tissue oxygen levels in the “lethal corner” should vary with activation level during normoxia are presented.

It is also interesting to note this work supports the conclusion of [55]: That in the physiological range, an increase in CBF is not required for the observed increase in CMRO_2_ to take place. In order to verify this, the full model was run with *R_u_* = 0 so that demand had no effect on blood flow. Again, significant increases in CMRO_2_ – up to about 45 percent – could occur. The relationship between oxygen levels and CMRO_2_ was also consistent with data in [55] as shown in Figure 8.

### Apparent K_m_ for O_2_ in the Simplified Model

Understanding the response of the ΔoxCCO signal to changes in oxygen concentration is central to understanding much experimental data. Yet the details of this response are controversial, even when measured during *in vitro* experiments in cells and mitochondria. Partly this arises from the technical difficulty of making measurements at low oxygen concentrations (see [56] for a lively discussion of this from one author). In particular, debate has centred around the K_m_ for oxygen consumption, which is known to be a complex function of cell metabolism [57]. Even simple models suggest that there is no need for standard Michaelis-Menten type behaviour of consumption rate with oxygen levels [58]. Apart from the uncertainties in the behaviour of consumption when oxygen concentration is dropped, there are also uncertainties about how mitochondrial redox states change in this situation. Again the quantitative response cannot be heuristically predicted, and there is contradictory data in the literature [59],[60].

**Figure 9:**
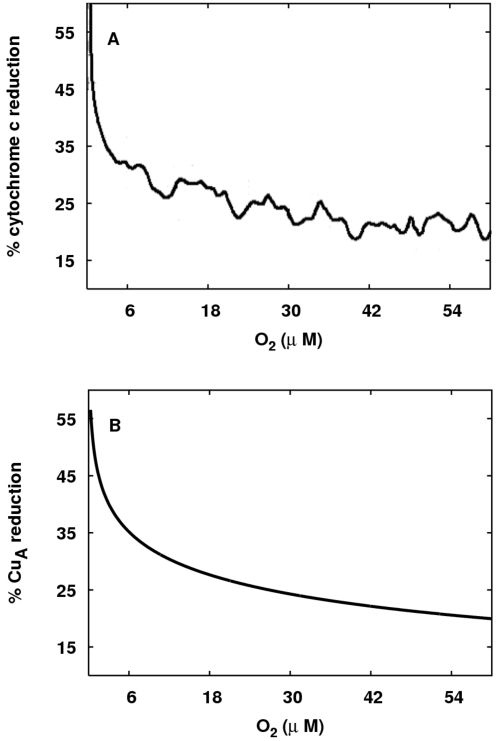
Comparison of experimentally measured and modelled CCO redox states. (A) How the level of reduction of cytochrome c varies with oxygen concentration (redrawn from Figure 5A of [20]). (B) The equivalent data for Cu_A_ from model simulations is presented. For the simulation, the reducing substrate is set to be succinate, and the demand parameter *u* is set to be low (*u* = 0.4) to represent a high phosphorylation potential.

We used our simplified model to explore some of these questions. There are very few reliable papers reporting on changes in the Cu_A_ redox state with oxygen; therefore we focussed on a key paper that reported on cytochrome c redox state changes [20], which we have shown is likely to be in close redox equilibrium with Cu_A_ during enzyme turnover [61]. Here we show that our model is capable of reproducing quantitatively key results from [20]. In Figure 9 the behaviour of redox state of cytochrome c and the equivalent data for Cu_A_ in the model are presented. There is good agreement between the experimental and modelled data. The figure caption gives details of the simulation.

**Figure 10:**
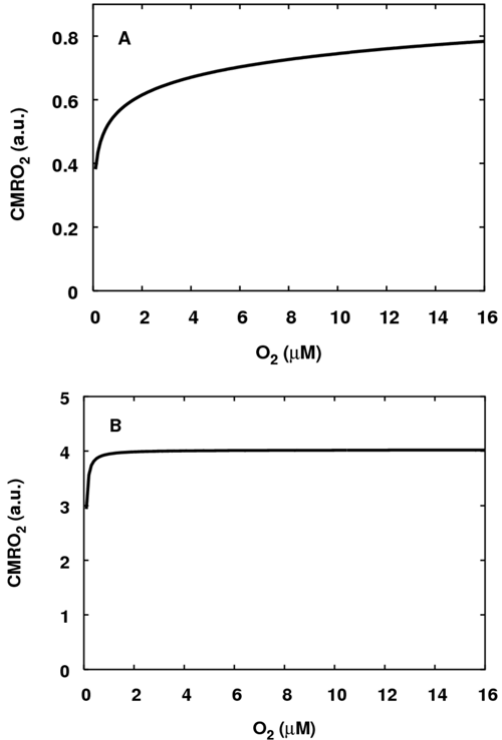
The response of steady state CMRO_2_ to a drop in mitochondrial O_2_ level. CMRO_2_ is in arbitrary units. (A) In coupled mitochondria. (B) Uncoupled mitochondria. As above, for both simulations, the reducing substrate is set to be succinate, so that input to the system is by electron transfer to ubiquinone, and the demand parameter *u* is set to be low (*u* = 0.4 in both simulations). For the uncoupled mitochondria, the parameter *k_unc_* is raised from its normal value of 1 to a value of 1000 giving an approximately four-fold increase in maximum CMRO_2_.

The apparent K_m_ for oxygen of mitochondrial oxygen consumption is quoted as 0.8 *μ*M in [33], consistent with values in [20]. The behaviour of CMRO_2_ as [O_2_] is lowered in the simplified model is illustrated in Figure 10. Details of the simulations are presented in the figure legend.

For the coupled mitochondria, half-maximal CMRO_2_ occurs at a little less than 1 *μ*M O_2_. For the uncoupled mitochondria half-maximal CMRO_2_ occurs below 0.1 *μ*M O_2_. (In order to calculate the Vmax—and hence K_m_—values in the case of the coupled mitochondria, larger values of oxygen than shown were needed. As with the model in [58], the graph does not fit a simple Michaelis-Menten curve well. In the uncoupled case the graph was blown-up for very low oxygen values in order to determine the K_m_ value.) The model values are consistent with the results in [20]. It should be noted that the low value of *u* (high phosphorylation potential) used in these simulations was essential to get the marked lowering of apparent K_m_ during uncoupling. Without this choice, the K_m_ for coupled mitochondria is also very low, suggesting that experimental results of this kind might be sensitive to experimental details such as the levels of ADP supplied.

In [58] we showed that the lowering of the K_m_ for oxygen during uncoupling can be achieved assuming that the effect of uncoupling is to inhibit the reverse reaction during which electrons are transferred from cyta3 to Cu_A_. However in the model presented in that paper the lowering in K_m_ was not accompanied by any increase in flux. As shown in the graphs above our new model can simultaneously achieve an increase in flux and a drop in the K_m_ for oxygen.

Obtaining the qualitative behaviour shown in Figure 9, the quantitative match in Figure 10, and the qualitative behaviour during functional activation in [5] and [24] was achieved by varying the six model parameters which control the response of reaction rates to Δp: i.e. Δp_30_, *c*3, *c_k_*_1_, *c_k_*_2_, *L_CV_*_,0_, *r_CV_* and Δp*_CV_*_0_. This is discussed further in Section C of Text S1.

### Behaviour of the Model during Hypoxia

As NIRS-derived parameters report on oxygen delivery and consumption in the brain, there is obviously wide interest in the effect of hypoxia on the NIRS signals. Indeed hypoxia is by far the most common *in vivo* NIRS challenge, especially in animal models. It is also amongst the most controversial, with different mathematical algorithms leading to different conclusions about the relationship between the haemoglobin-based NIR signals and that of ΔoxCCO [22], [62]–[64]. Even with a single algorithm [65] different physiological explanations have been proposed for the changes during hypoxia (large decrease in oxCCO from baseline) and immediately post-hypoxia (small increase in oxCCO from baseline).

Currently the debates in this area have revolved around the physics of making the measurements (choice of wavelengths, effect of multiple tissue layers on light transport, etc.) Moreover, the systems studied have not always been identical (animal models versus humans and newborn versus mature), raising the possibility of differences in the underlying biochemistry and physiology. Therefore an analysis of how our model behaves during hypoxia, and how variations in the model parameters affect the relationship between the NIR signals, is clearly important, being independent of measurement concerns and allowing an exploration of possible effects of physiological variation.

**Figure 11:**
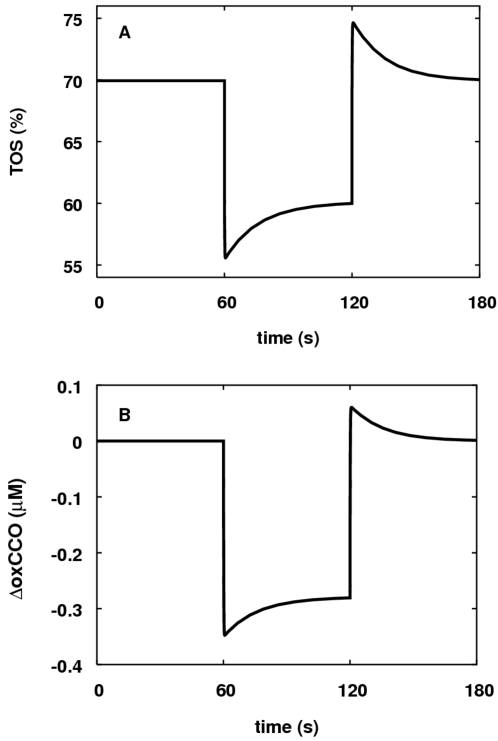
Model response of TOS and ΔoxCCO to a step down in arterial oxygen saturation. (A) Response of TOS (percent). (B) Response of ΔoxCCO (*μ*M). A hyperaemic effect is seen in both signals.

The dynamic and steady state responses of modelled NIRS signals to hypoxia were explored. In the first simulation a one minute drop in arterial oxygen saturation from 96 percent to 80 percent was carried out. The results are plotted in Figure 11. Following hypoxia there is an increase in blood flow leading to a partial restoration of TOS (and to a lesser extent ΔoxCCO) during the hypoxia. This behaviour is connected with the rapidity of the drop in arterial oxygen saturation and so in simulations of real hypoxias (see next section) this adaptation is unlikely to be observed. Both TOS and ΔoxCCO show an overshoot associated with the hyperaemia following reoxygenation, consistent with some experimental observations [65].

**Figure 12:**
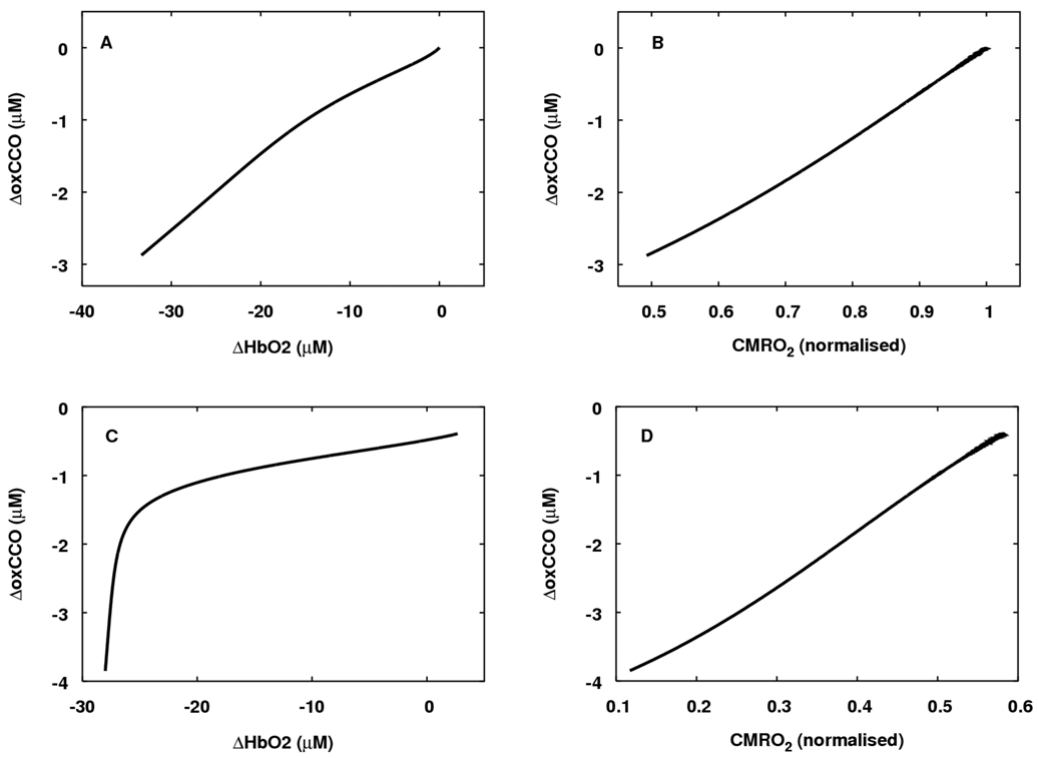
Relationship between ΔHbO2, ΔoxCCO and CMRO_2_ during changes in arterial oxygen saturation. (A) The model was run with normal parameter values and an approximately linear relationship between ΔHbO2 and ΔoxCCO held. (B) At these same normal parameter values CMRO_2_ showed an approximately linear relationship with ΔoxCCO. (C) Baseline CMRO_2_ was lowered to about 60 percent of the normal model baseline, by setting *u* = 0.1, while normal CBF was also lowered by about the same amount by setting CBF_n_ = 0.007 ml blood per ml brain tissue per second. A more clearly biphasic relationship between ΔHbO2 and ΔoxCCO was obtained. (D) Again, at the changed parameter values, CMRO_2_ had an approximately linear relationship with ΔoxCCO.

In [66] data on the relationship between ΔHbO2 and ΔoxCCO during hypoxia is presented. In order to test the model behaviour in this situation, a steady state simulation (as in the production of steady state curves above) was carried out. The results of this simulation are plotted in Figure 12.

In [66] a very clear biphasic relationship was reported between ΔHbO2 and ΔoxCCO. At normal parameter values, although the model does predict increased sensitivity of ΔoxCCO to oxygen levels at lower oxygen levels, the biphasic relationship is slight (Figure 12A). Interestingly, lowering both demand (and hence baseline CMRO_2_) and normal blood flow leads to a considerably more marked nonlinearity in the relationship (Figure 12C). This simultaneous change in demand and normal flow leads to a normal TOS of about 60 percent consistent with that calculated from the absolute oxy- and deoxy-haemoglobin values in [66].

This leads to some interesting questions. In both of the simulations above, ΔoxCCO has an approximately linear relationship with CMRO_2_ (Figure 12B and 12D), and so any significant drop in ΔoxCCO implies that arterial oxygen supply can no longer match demand – an event we can term metabolic failure. The simulations indicate that the threshold for metabolic failure can be more or less sharp depending on the normal matching of oxygen supply and demand for an individual. They raise the possibility that the relationship between ΔHbO2 and ΔoxCCO during hypoxia may depend on differences between species, age, and possibly individual, with some individuals being more vulnerable to hypoxia. This may have important implications for clinical management of patients in neurocritical care.

### Comparison with *In Vivo* Data

**Figure 13:**
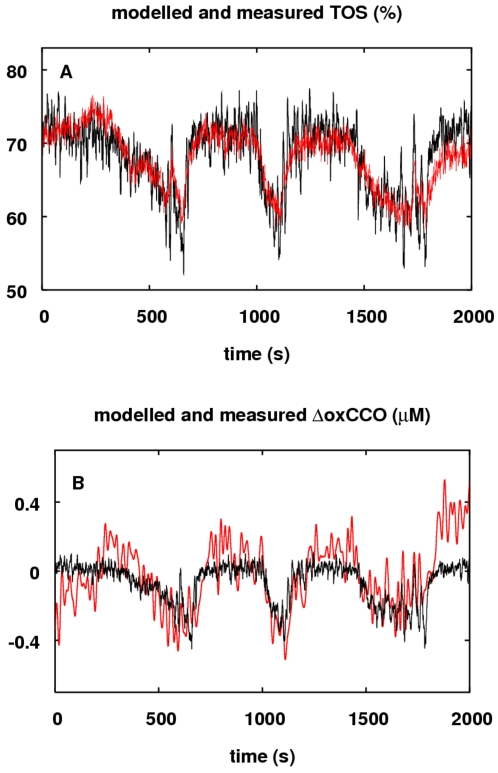
Responses of measured and modelled TOS and ΔoxCCO during a hypoxia challenge. Measured (red) and modelled (black) responses of (A) TOS (%) and (B) ΔoxCCO (*μ*M) are shown. Details are given in the text.

In the future we intend to challenge our model to reproduce a wide variety of *in vivo* data sets. Here we present preliminary results in this direction. First we compared our model output to experimental data from subjects undergoing the most common challenge used to provoke responses in the oxCCO signal – cerebral hypoxia. The data is from a study described in [67]. Modelled and measured TOS and ΔoxCCO signals for a subject undergoing a hypoxic challenge are presented in Figure 13.

The stimuli were a series of drops in inspired oxygen and consequent drops in arterial oxygen saturation. Experimentally measured inputs to the model were SaO2, PaCO2 and mean arterial blood pressure. All inputs were down-sampled to 1 Hz. The baseline value of the ΔoxCCO signal has been brought to zero, and in order to remove high frequency noise the data has been filtered using a 5th order low pass Butterworth filter with a cut-off frequency of 0.1 Hz (Matlab Mathworks Inc.)

In spite of the known inter-subject and regional variability in TOS, both baseline TOS and changes in TOS are predicted well for this subject by the model. The model seems to slightly underestimate ΔoxCCO signal changes, although given the level of noise in the experimental data the extent of this is not clear.

**Figure 14:**
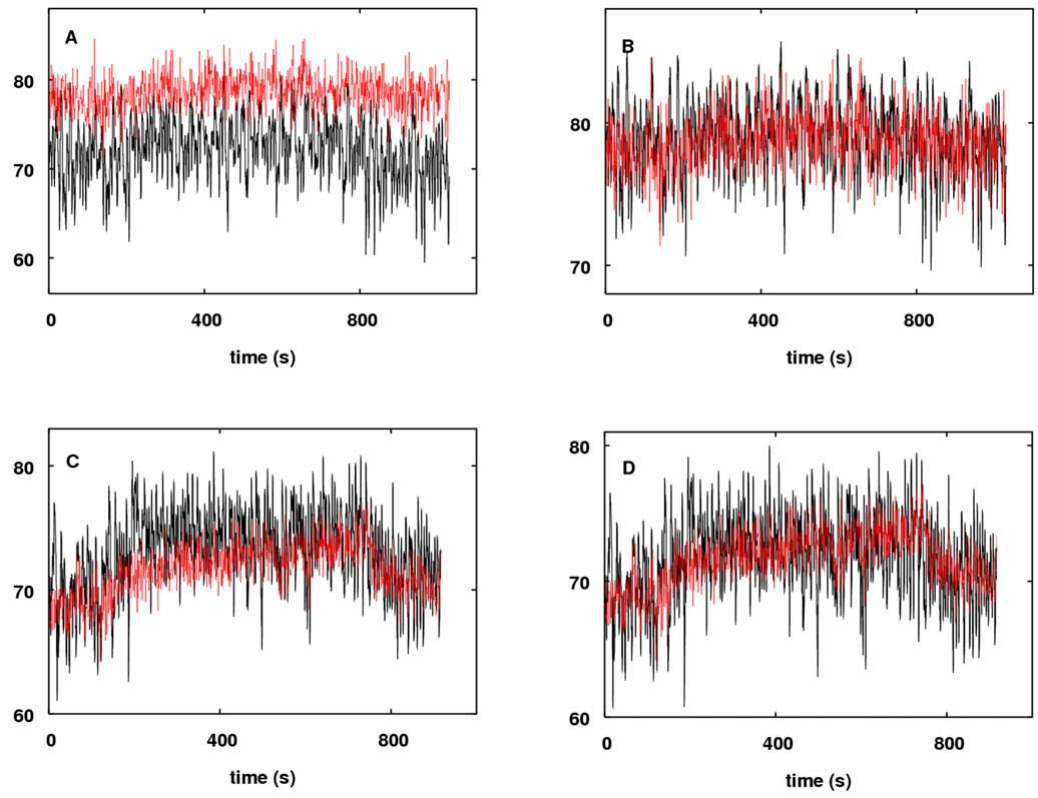
Responses of measured and modelled TOS during a hypercapnia challenge. Measured (red) and modelled (black) responses of TOS: (A) For subject 1 without optimisation. (B) For subject 1 following optimisation of AVRn and *R_C_*, which gave values of AVRn = 1.28 and *R_C_* = 1.31. (C) For subject 2 without optimisation. (D) For subject 2 following optimisation of AVRn and *R_C_*, which gave values of AVRn = 0.286 and *R_C_* = 1.62.

As a test of the model's behaviour in the context of changes in arterial CO_2_, NIRS data from healthy subjects monitored while undergoing moderate hypercapnia, described in [68], was compared with model predictions. In this study, the only NIRS signal monitored was TOS. There was wide variation in baseline TOS between subjects, corresponding to natural variability in blood flow and CMRO_2_, but more importantly to the fact that the arterio-venous ratio in the region of tissue queried can have high variability. In all cases the modelled and measured data were qualitatively comparable before any attempt to optimise model parameters. However a good fit to the data could be obtained by varying two parameters: Normal arterio-venous ratio AVRn, and *R_C_*, the sensitivity of blood flow to PaCO2. Despite the fact that information is often *not* clearly visible in the data (see Figure 14A, for example), in all cases but one, optimisation gave positive values for *R_C_*, in other words, the model was able to detect a positive cerebrovascular reactivity to CO_2_ in the data—a fact which is potentially of clinical importance ([69] for example). Two examples of data-sets before and after fitting are presented in Figure 14.

Overall, preliminary comparisons between modelled and measured *in vivo* data are encouraging. A future task will be to compare further data from these studies and other *in vivo* studies with model outputs.

### Conclusions and Future Work

A basic model of the control of cerebral blood flow and the behaviour of various NIRS signals has been presented. The model is relatively simple, containing very few dynamic variables, but nevertheless preliminary simulations show that it is capable of reproducing basic expected behaviours, and matching experimentally measured data. One important conclusion from these simulations is that the ΔoxCCO signal contains information above and beyond what is available from the other NIRS signals. This in turn gives more hope of achieving the ultimate aim: Real time reconstruction from NIRS data of underlying physiological events of clinical importance.

So far, several model parameters have only been set heuristically, and comparison with measured data has not been systematic. The immediate next stage is to explore systematically the effects of model parameters on important model behaviours, for example on the K_m_ for oxygen during hypoxia and the direction of the ΔoxCCO signal during activation. Once key outputs are identified it will be possible to carry out a sensitivity analysis of the kind carried out in [34]. Parallel to identifying how model behaviour is sensitive to parameter values, is the need to identify which parameters are liable to show variability between individuals, or between health and pathology. Some of our observations in these directions are presented in Text S1. Once these parameters have been identified, optimisation of the kind described in Figure 14 can focus on setting these parameters from an individual's data.

A number of limitations of the model have been pointed out in the text. The limitations we consider most serious are:

- The treatment the vascular tree via a single typical radius, and of venous volume as fixed: These simplifications were based on the ability of one-compartment models to reproduce data in [44],[45] and [48] and on the relatively small changes in venous volume during simulations of the model in [29]. These approximations might cause some error in predictions of NIRS oxy- and deoxy-haemoglobin levels.
- The treatment of regulatory stimuli as additive in a simplistic way, and each with a single time constant, hides the complexity described in [32].
- The treatment of demand via a single parameter *u*. If this parameter is related to the phosphorylation potential, then we would expect it not to be a control parameter, but rather itself to be affected by events such as changes in oxygenation, introducing additional feedbacks into the model.
- The treatment of the final transfer of electrons to oxygen as a single step: Given the complexity of events following oxygen binding to cytochrome *c* oxidase [70] this might introduce incorrect behaviour into the model.
- The setting of some model parameters in heuristic ways because of insufficient data to ensure accurate setting.

By running sensitivity analyses and comparisons with experimental data it will become clear which of these limitations affect model behaviour appreciably, enabling us to refine the model as necessary. The process of gathering data needed to help validate the model is ongoing. Once the model is well validated it should be possible to integrate its use into the normal NIRS measurement process, greatly enriching the value of the measured data.

## Supporting Information

Text S1 Supplementary material
(0.21 MB PDF)
