# Supplementary material for: A Model of Brain Circulation and Metabolism: NIRS Signal Changes during Physiological Challenges
Source: PLoS Comput Biol. 2008 Nov 7;4(11):e1000212. doi: 10.1371/journal.pcbi.1000212 (PMC2573000; doi:10.1371/journal.pcbi.1000212)
Supplement: Text S1 — Supplementary material (0.21 MB PDF) [file pcbi.1000212.s001.pdf]

Supplementary material for "A model of brain circulation and metabolism"

A Model equations

Apart from the equations defining the first-order filtered regulatory stimuli, v_{P_a}, v_{O_2}, v_{PaCO2} and v_u, the model has only five dynamic variables, $\Delta\Psi$ and the four mitochondrial concentrations: CuA_o, $a3_r$, [H], and $[O_2]$. A minimal set of equations describing the model are provided below:

$$\frac{dv_x}{dt} = \frac{1}{\tau_x}(x - v_x), \qquad x = P_a, O_2, PaCO2, u \tag{A.1}$$

$$\frac{d\,CuA_o}{dt} = 4(f_2 - f_1) \tag{A.2}$$

$$\frac{d\,a3_r}{dt} = 4(f_2 - f_3) \tag{A.3}$$

$$\frac{d\,[H]}{dt} = (-p_1 f_1 - p_2 f_2 - p_3 f_3 + L)/\mathrm{Vol_{Hi}} \tag{A.4}$$

$$\frac{d\,\Delta\Psi}{dt} = \frac{p_1 f_1 + p_2 f_2 + p_3 f_3 - L}{C_{im}} \tag{A.5}$$

$$\frac{d\,[O_2]}{dt} = J_{O2}/\mathrm{Vol_{mit}} - f_3 \tag{A.6}$$

$$f_1 = k_{1,n} \frac{NADH}{NADH_n} \exp(-c_{k1}(\Delta p - \Delta p_n)) \left[CuA_o - \frac{CuA_r}{10^{-(p_1 \Delta p/4 - E_1(NADH))/Z}}\right] \tag{A.7}$$

$$f_2 = k_{2,n} \exp(-c_{k2}(\Delta p - \Delta p_n)) \left[CuA_r\, a3_o - \frac{CuA_o\, a3_r}{10^{-(p_2 \Delta p/4 - E_2)/Z}}\right] \tag{A.8}$$

$$f_3 = k_{3,0}[O_2]a3_r \frac{\exp(-c3\Delta p)(1 + \exp(c3\,\Delta p_{30}))}{1 + \exp(-c3(\Delta p - \Delta p_{30}))} \tag{A.9}$$

$$L = L_{CV,max} \left(\frac{1 - \exp[-k_{CV}(\Delta p - \Delta p_{CV0} + Z\ln(u))]}{1 + r_{CV}\exp[-k_{CV}(\Delta p - \Delta p_{CV0} + Z\ln(u))]}\right) + k_{unc}L_{lk0}(\exp(k_{lk2}\Delta p) - 1) \tag{A.10}$$

$$\mathrm{Vol_{Hi}} = \frac{1000\ C_{buffi}\ \mathrm{Vol_{mit}}\ dpH}{[H]\ (1 - 10^{-dpH})} \tag{A.11}$$

$$J_{O2} = \min\{D_{O2}([O_{2,c}] - [O_2]),\ CBF[HbO_{2,a}]\} \quad \text{with smooth approximation} \tag{A.12}$$

$$J_{O2} = c - \frac{\sqrt{(x+c)^2 + \epsilon^2} - (x+c)}{2} \quad \text{where}$$
$$c = CBF[HbO_{2,a}],\ \ x = D_{O2}([O_{2,c}] - [O_2]),\ \ \epsilon = CBF_n[HbO_{2,a,n}]/10 \tag{A.13}$$

$$CBF = K_G(P_a - P_v)r^4 \tag{A.14}$$

$$[\mathrm{O_{2,c}}] = \phi \left(\frac{2\mathrm{SaO2} - J_{\mathrm{O2}}/(\mathrm{CBF}\ [\mathrm{Hbtot}])}{2 + J_{\mathrm{O2}}/(\mathrm{CBF}\ [\mathrm{Hbtot}])} \right)^{\frac{1}{n_h}} \tag{A.15}$$

$$r = \frac{(\sigma_{e0}(\exp(K_\sigma(r - r_0)/r_0) - 1) - \sigma_{coll})h + T_{max0}(1 + k_{aut}\mu) \exp\left(-\left|\frac{r - r_m}{r_t - r_m}\right|^{n_m}\right)}{(P_a + P_v)/2 - P_{ic}} \tag{A.16}$$

$$h = \sqrt{r^2 + (r_0 + h_0)^2 - r_0^2} - r \tag{A.17}$$

$$\mu = \frac{\mu_{min} + \mu_{max} e^\eta}{1 + e^\eta} \tag{A.18}$$

$$\eta = R_P \left(\frac{v_{P_a}}{v_{P_a,n}} - 1 \right) + R_O \left(\frac{v_{\mathrm{O_2}}}{v_{\mathrm{O_2},n}} - 1 \right) + R_C \left(1 - \frac{v_{\mathrm{PaCO2}}}{v_{\mathrm{PaCO2},n}} \right) + R_u \left(1 - \frac{v_u}{v_{u,n}} \right). \tag{A.19}$$

In the case of the simplified model Equations A.1, A.6 and A.12-A.19 are omitted, mitochondrial oxygen $[\mathrm{O_2}]$ is a controllable parameter, and f_1 (Equation A.7) takes the form

$$f_1 = \mathrm{k_{1,n}} \frac{\mathrm{UQH_2}}{\mathrm{UQH_{2,n}}} \exp(-c_{k1}(\Delta\mathrm{p} - \Delta\mathrm{p}_n)) \left[\mathrm{CuA_o} - \frac{\mathrm{CuA_r}}{10^{-(p_1 \Delta\mathrm{p}/4 - E_1(\mathrm{UQH_2}))/Z}} \right]. \tag{A.20}$$

Note that several of the equations are implicit or need to be solved simultaneously. Apart from CBF above, important model output variables (or observables), which can potentially be used to compare model behaviour to measured quantities *in vivo* are

$$\mathrm{SvO2} = \mathrm{SaO2} - \frac{J_{\mathrm{O2}}}{\mathrm{CBF}[\mathrm{Hbtot}]} \tag{A.21}$$

$$\mathrm{CMRO_2} = \mathrm{Vol_{mit}} f_3 \tag{A.22}$$

$$\mathrm{TOS} = \frac{\mathrm{AVRn}(r/r_n)^2 \mathrm{SaO2} + \mathrm{SvO2}}{(\mathrm{AVRn}(r/r_n)^2 + 1)} \tag{A.23}$$

$$\Delta\mathrm{Hbt} = \frac{1000}{4} \left(\mathrm{Vol_{art,n}} \left(\frac{r}{r_n} \right)^2 + \mathrm{Vol_{ven}} \right) [\mathrm{Hbtot}] \mathrm{Vol_{blood,n}} - \mathrm{Hbt_n} \tag{A.24}$$

$$\Delta\mathrm{HbO2} = \frac{1000}{4} \left(\mathrm{Vol_{art,n}} \left(\frac{r}{r_n} \right)^2 \mathrm{SaO2} + \mathrm{Vol_{ven}} \mathrm{SvO2} \right) [\mathrm{Hbtot}] \mathrm{Vol_{blood,n}} - \mathrm{HbO2_n} \tag{A.25}$$

$$\Delta\mathrm{HHb} = \mathrm{Hbt} - \mathrm{HbO2} - \mathrm{HHb_n} \tag{A.26}$$

$$\Delta\mathrm{oxCCO} = 1000\, \mathrm{Vol_{mit}} \left(\mathrm{CuA_o} - \mathrm{CuA_{o,n}} \right). \tag{A.27}$$

B Glossary of model variables

Where concentrations are given units of mM without further characterisation, the reference volume is that of the compartment in which the quantity resides. Where there is ambiguity the volume in question is made explicit. In the case of NIRS quantities, unit conversions are carried out to give concentrations in micromoles per unit tissue volume. Initialisation is only needed for differential variables.

variable	brief description	units	initialisation
**Differential variables**			
$\mathrm{CuA_o}$	oxidised $\mathrm{Cu_A}$ concentration	mM	$\mathrm{CuA_{o,n}}$
$\mathrm{a3_r}$	reduced cyt $\mathrm{a_3}$ concentration	mM	$\mathrm{a3_{r,n}}$
[H]	mitochondrial proton concentration	mM	$1000(10^{-\mathrm{pH_{m,n}}})$
$\Delta\Psi$	mitochondrial inner membrane potential	mV	$\Delta\Psi_n$
$[\mathrm{O_2}]$	mitochondrial oxygen concentration	mM	$[\mathrm{O}_{2,n}]$
v_{P_a}	first-order filtered P_a	mmHg	$P_{a,n}$
$v_{\mathrm{O_2}}$	first-order filtered $[\mathrm{O_2}]$	mM	$[\mathrm{O}_{2,n}]$
v_{PaCO2}	first-order filtered PaCO2	mmHg	$\mathrm{PaCO2_n}$
v_u	first order filtered u	none	1
**General**			
$\mathrm{CMRO_2}$	cerebral metabolic rate of oxygen consumption	mmol (l tissue)$^{-1}$ s^{-1}	–
J_{O2}	rate of oxygen flux	mmol (l tissue)$^{-1}$ s^{-1}	–
TOS	absolute tissue oxygen saturation	none	–
ΔoxCCO	changes in tissue concentration of oxidised $\mathrm{Cu_A}$	μmol (l tissue)$^{-1}$	–
ΔHHb	changes in tissue concentration of deoxyhaemoglobin	μmol (l tissue)$^{-1}$	–
ΔHbO2	changes in tissue concentration of oxyhaemoglobin	μmol (l tissue)$^{-1}$	–
ΔHbt	changes in tissue concentration of total haemoglobin	μmol (l tissue)$^{-1}$	–

ctd...

variable	brief description	units	initialisation
**Blood chemistry and blood flow regulation**			
SvO2	oxygen saturation of venous blood	none	–
ScO2	typical oxygen saturation of capillary blood	none	–
CBF	cerebral blood flow	ml blood (ml tissue)$^{-1}$s^{-1}	–
r	typical vascular radius	cm	–
h	vascular wall thickness	cm	–
η	weighted sum of regulatory stimuli	none	–
μ	level of regulatory input	none	–
G	conductance of the cerebral circulation	ml blood (ml tissue)$^{-1}$ s^{-1} mmHg^{-1}	–
σ_e	elastic stress in vessel wall	mmHg	–
T_e	elastic tension in vessel wall	mmHg cm	–
T_m	muscular tension in vessel wall	mmHg cm	–
T_{max}	maximum muscular tension in vessel wall	mmHg cm	–
**Mitochondria**			
$\mathrm{CuA_r}$	reduced $\mathrm{Cu_A}$ concentration	mM	–
$\mathrm{a3_o}$	oxidised cyt $\mathrm{a_3}$ concentration	mM	–
$\Delta\mathrm{p}$	proton motive force	mV	–
$\mathrm{pH_m}$	mitochondrial pH	pH units	–
$\mathrm{Vol_{art}}$	arterial volume as a fraction of normal total blood volume	none	–
$\mathrm{Vol_{Hi}}$	effective fractional mitochondrial volume for protons	none	–
f_1	rate of reaction 1	mM s^{-1}	–
f_2	rate of reaction 2	mM s^{-1}	–
f_3	rate of reaction 3	mM s^{-1}	–

ctd...

variable	brief description	units	initialisation
ΔG_1	free energy associated with reaction 1	mV	–
ΔG_2	free energy associated with reaction 2	mV	–
k_1	forward rate constant for reaction 1	s^{-1}	–
k_{-1}	backward rate constant for reaction 1	s^{-1}	–
Keq_1	equilibrium constant for reaction 1	none	–
k_2	forward rate constant for reaction 2	$mM^{-1}s^{-1}$	–
k_{-2}	backward rate constant for reaction 2	$mM^{-1}s^{-1}$	–
Keq_2	equilibrium constant for reaction 2	none	–
L	rate of proton re-entry into mitochondrial matrix	$mM\ s^{-1}$	–
L_{CV}	rate of proton re-entry via ATP synthase and associated processes	$mM\ s^{-1}$	–
L_{lk}	rate of proton re-entry via leak channels	$mM\ s^{-1}$	–

variable table ends.

C Parameter setting

Many parameters in the model are set with reference to other parameters, or in order to ensure correct "normal" behaviour. Others are directly given values. Throughout, the subscript n indicates a normal value of some variable or control parameter.

C.1 A table of model parameters with numerical values

Where no units are given, this is because the parameter in question is a dimensionless quantity.

parameter	brief description	value and units	references
**Blood chemistry, blood flow regulation and volume**			
$\mathrm{PaCO2_n}$	normal arterial partial pressure of CO_2	40 mmHg	[1]
PaCO2	arterial partial pressure of CO_2	40 mmHg	[1][a]
$\mathrm{SaO2_n}$	normal saturation of the arterial haemoglobin	0.96	[b]
SaO2	saturation of arterial haemoglobin	0.96	[a,b]
[Hbtot]	Total haemoglobin concentration in the arteries and veins.	9.1 mM	[2][a,b]
$[\mathrm{Hbtot_n}]$	Normal total haemoglobin concentration in the arteries and veins.	9.1 mM	[2][b]
P_a	arterial blood pressure	100 mmHg	[1][a]
$P_{a,n}$	Normal value of ABP	100 mmHg	[1][b]
P_v	venous blood pressure	4 mmHg	[1][a]
$P_{v,n}$	normal venous blood pressure	4 mmHg	[1][b]
P_{ic}	intracranial blood pressure	9.5 mmHg	[1][b]
R_C	sensitivity of η to PaCO2	2.2	[c]
R_O	sensitivity of η to $[O_2]$	1.5	[d,f]
R_P	sensitivity of η to arterial pressure	4.0	[e]
R_u	parameter controlling sensitivity of η to u	0.5	[d,f]
τ_{P_a}	the time constant associated with v_p	5 s	[j]

ctd...

parameter	brief description	value and units	references
τ_{CO_2}	the time constant associated with v_c	5 s	[j]
τ_{O_2}	the time constant associated with v_o	20 s	[j]
τ_u	the time constant associated with v_u	0.5 s	[j]
k_{aut}	control parameter allowing destruction of autoregulation	1	[a]
σ_{coll}	parameter in the pressure-elastic tension relationship	62.79 mmHg	[1]
σ_{e0}	parameter in relationship determining σ_e	0.1425 mmHg	[1]
K_σ	parameter controlling sensitivity of σ_e to radius	10 mmHg	[1]
n_m	exponent in the muscular tension relationship	1.83	[1]
r_m	value of vessel radius giving maximum muscular tension	0.027 cm	[1]
r_t	parameter in the muscular tension relationship	0.018 cm	[1]
h_0	vascular wall thickness when radius is r_0	0.003 cm	[1]
r_0	radius in the elastic tension relationship	0.0126 cm	[h]
r_n	normal radius of blood vessels	0.0187 cm	[h]
$\mathrm{CBF_n}$	normal cerebral blood flow	0.01075 ml blood (ml tissue)$^{-1}$ s^{-1}	[b,f]
AVRn	The normal arterio-venous volume ratio	0.333	[a,f]
$\mathrm{Vol_{blood,n}}$	normal blood volume as a fraction of brain volume	0.04	[3][f]
**Oxygen transport and consumption**			
$\mathrm{CMRO_{2,n}}$	normal $\mathrm{CMRO_2}$	0.034 mmol (l tissue)$^{-1}$ s^{-1}	[4][f]

ctd...

parameter	brief description	value and units	references
$[O_{2,n}]$	normal oxygen concentration in mitochondria	0.024 mM	[5, 6][b]
ϕ	value of $[O_2]$ at half maximal saturation	0.036 mM	[7][f]
n_h	hill coefficient for haemoglobin saturation	2.5	[7]
**Mitochondria**			
C_{im}	Capacitance of mitochondrial inner membrane	0.00675 mmol $(\text{l mito})^{-1}$ mV^{-1}	[8]
D_{NADH}	a parameter controlling the effect of activation on glycolytic/TCA cycle flux	0.01	[d,f]
$L_{lk,frac}$	normal fraction of proton entry into mitochondria which is via leak channels	0.25	[9][f]
Vol_{mit}	mitochondrial volume fraction	0.067	[10]
Z	standard physico-chemical constant $(2.303RT/F)$	59.028 mV	[11]
$\Delta\Psi_n$	normal mitochondrial inner membrane potential	145 mV	[12]
$\mathcal{E}_0(\text{NADH})$	NADH standard redox potential	-320 mV	[12]
$\mathcal{E}_0(\text{UQ})$	ubiquinone standard redox potential	60 mV	[12]
$\mathcal{E}_0(\text{Cu}_\text{A})$	CuA standard redox potential	247 mV	[13][f]
$\mathcal{E}_0(\text{cyt a}_3)$	cyt a3 standard redox potential	350 mV	[14, 15] [f]
$\text{CuA}_{\text{frac,n}}$	normal oxidised fraction of CuA	0.8	[16][f]
N_t	total mitochondrial NAD pool	3 mM	[8, 10]
$\text{N}_{\text{rat,n}}$	normal mitochondrial NAD/NADH ratio	9	[17–19][f]
U_t	total mitochondrial ubiquinone pool	1.35 mM	[8, 10]
U_{rat}	UQ/UQH_2 ratio	1	[a,f]
$\text{U}_{\text{rat,n}}$	normal UQ/UQH_2 ratio	1	[f]

ctd...

parameter	brief description	value and units	references
$\mathrm{cytox_{tis}}$	concentration of cytochrome c oxidase in tissue	0.0055 mmol (l tissue)$^{-1}$	[20]
$\mathrm{pH_o}$	extra-mitochondrial pH	7.0	[10][a]
$\mathrm{pH_{o,n}}$	normal extra-mitochondrial pH	7.0	[10]
$\mathrm{pH_{m,n}}$	normal mitochondrial pH	7.4	[11][f]
dpH	constant in the pH buffering relationship	0.001	[10]
$\mathrm{C_{buffi}}$	buffering capacity for protons in mitochondria	0.022 M H^+/pH	[11]
$\Delta \mathrm{p}_{CV0}$	A constant in the rate of complex V	90 mV	[f,g]
r_{CV}	parameter controlling the ratio of maximal to minimal rates of oxidative phosphorylation	5	[f,g]
$L_{CV,0}$	normal complex V flux as a fraction of maximum possible flux	0.4	[f,g]
$\Delta \mathrm{p}_{30}$	value of Δp at which reaction 3 is maximally sensitive to Δp	143.61 mV	[f,g]
c_{k1}	parameter controlling sensitivity of k_1 to PMF	0.01	[g]
c_{k2}	parameter controlling sensitivity of k_2 to PMF	0.02	[21][f,g]
$c3$	parameter controlling the sensitivity of reaction 3 to PMF	0.11	[g]
$\mathrm{k_{3,0}}$	apparent second-order rate constant for reaction 3 at $\Delta \mathrm{p} = 0$	2.5×10^5 mM^{-1} s^{-1}	[22]
k_{lk2}	constant controlling rate L_{lk}	0.038 mV^{-1}	[10]
k_{unc}	parameter representing the action of uncouplers	1	[a]
p_{tot}	protons pumped by chain	20 (12 in simplified model)	[9, 23][f]
p_1	protons pumped by complexes I-III	12 (4 in simplified model)	[9, 23][f]

ctd...

parameter	brief description	value and units	references
p_2	protons pumped by complex IV during oxidative phase	4	[9, 23][f]
p_3	protons pumped by complex IV during reductive phase	4	[9, 23][f]

parameter table ends.

[a]Control parameter. See discussion below.
[b]Typical value with wide range in normal variation.
[c]Fit to data from [24].
[d]Set heuristically: The parameter is needed in the model, but we have not yet gathered and analysed sufficient data to set it accurately.
[e]Set from data in [25, 26] as described in the text.
[f]Further discussion in the text.
[g]The parameters Δp_{30}, $c3$, c_{k1}, c_{k2}, $L_{CV,0}$, r_{CV} and Δp_{CV0} have collectively been set to give the behaviour shown in Figures 9 and 10 in the main text, and to give the qualitative behaviour during functional activation in [27] and [28]. They have not been optimised to fit any particular data-set.
[h]The parameters r_n, r_0 and R_P are set following the methodology described in Section E below.
[j]Accurate setting of the time constants requires data with good time resolution gathered from studies in the contexts of hypoxia, hypercapnia, changes in mean arterial blood pressure and functional activation. Currently these are set heuristically.

C.2 Control parameters

Several model parameters are control parameters, i.e. parameters whose values are expected to vary between experimental contexts and which will ideally be set from measured data for a particular experiment, or varied to optimise a fit to data.

The four control parameters P_a, PaCO2, SaO2 and u and are set to their normal values of 100 mmHg, 40 mmHg, 0.96 (i.e. 96 percent), and 1 respectively. The first three are often monitored in clinical/experimental situations where NIRS signals are measured, and these measured inputs would then be used as inputs to the model. The values above are used as the defaults in the absence of such input data. u on the other hand – representing the level of neuronal activation – is assumed to be constant at baseline value except in the simulations of functional activation.

Central venous pressure P_v is rarely monitored in clinical/experimental situations where NIRS signals are measured, and so is assumed to remain at its normal value of 4 mmHg.

Hbtot can be considered a control parameter as it is likely to show considerable physiological variation. However for all simulations in this paper, it is set to its default value $\mathrm{Hbtot_n}$.

k_{aut} has not been changed from its normal value of 1 (full autoregulation) in any simulations presented here. However, we anticipate that it may be settable from experimental data for patients who may have impaired autoregulation.

k_{unc} was varied from its normal value of 1 in simulations designed to compare model behaviour to data reported in [29].

U_{rat} is needed only in simulations of the simplified model and its value depends on experimental context. In the simulations of data from [29] we have used a value of 1. This parameter and $U_{rat,n}$ are discussed further below.

C.3 Blood flow and volume

Directly set parameters

$P_{v,n} = 4$ mmHg, $P_{ic} = 9.5$ mmHg are taken from [1].

AVRn = 1/3. This value sets normal arterial blood volume as one quarter of total blood volume. We expect this quantity to show significant physiological variation, and in the case of NIRS signals to show some regional variation, giving rise to different baseline levels of TOS. The value of 1/3 has been set in order to give baseline TOS of approximately 70 % in line with various data sets, for example the data described in [30].

$Vol_{blood,n} = 0.04$. Blood volume is given as 2-5 percent of tissue volume on p15 of [3]. Similar estimates can be found in other sources. This parameter is important in determining the relative sizes of TOS and HbO2 changes.

$CBF_n = 0.01075$ ml blood per ml brain tissue per second is the normal value of CBF consistent with the consensus values of 750-1000 ml blood per minute and a normal brain volume of about 1400 ml.

$n_m = 1.83$, $r_t = 0.018$ cm, $r_m = 0.027$ cm, $h_0 = 0.003$ cm, $\sigma_{e0} = 0.1425$ mmHg, $K_\sigma = 10.0$ and $\sigma_{coll} = 62.79$ mmHg are all values for the proximal arterial segment in [1].

$r_n = 0.0187$ cm. r_n is simply a typical radius whose value can be normalised to 1 provided that all other quantities associated with the vessel (r_t, h_0, r_m and r_0) are appropriately rescaled. However, as many of the parameters connected with biophysical properties of the vessel walls are taken from the model in [1], a value intermediate between the normal values for the proximal and distal segments in this model (from model simulations) is chosen.

$r_0 = 0.0126$ cm is somewhat lower than the value for the proximal arterial segment in [1], and is chosen to give a reasonable autoregulation curve in a one-compartment model. A more complete discussion can be found in Section E below.

$\mu_{min} = -1$ so that maximal dilation occurs when there is no smooth muscle tension. $\mu_{max} = 1$, so that normal maximal smooth muscle tension is half of the maximum.

$R_P = 4.0$, $R_O = 1.5$, $R_u = 0.5$ and $R_C = 2.2$ are all chosen in order to reproduce standard responses

to the different stimuli. Discussion of R_P can be found in Section E below. R_C has been set to fit data in [24]. The value of R_u has been chosen to give results consistent with the blood flow to $CMRO_2$ ratio in [31]. [32] and the references therein suggest a lower value. R_O has been set to allow a decrease of a few percent in CBF during hyperoxia and an approximate doubling of CBF during severe hypoxia. The details of the response of CBF to hypoxia show considerable variability [33].

The response times $\tau_{P_a} = 5$ s, $\tau_{O_2} = 20$ s, $\tau_{CO_2} = 5$ s and $\tau_u = 0.5$ s are currently set heuristically. We anticipate that examination of well time-resolved data can be used to refine these quantities.

Calculated parameters

Normal conductance is set from normal flow and normal pressure difference:

$$G_n = \mathrm{CBF_n}/(P_{a,n} - P_{v,n}).$$

K_G (ml blood (ml brain tissue)$^{-1}$ s^{-1} mmHg^{-1} cm^{-4}) is determined from r_n and G_n:

$$K_G = G_n/r_n^4 \,.$$

Normal vessel wall thickness is set from normal vessel radius via:

$$(r_n + h_n)^2 - r_n^2 = (r_0 + h_0)^2 - r_0^2 \,.$$

Normal elastic tension $T_{e,n}$ is set by:

$$T_{e,n} = (\sigma_{e0}(e^{K_\sigma(r_n - r_0)/r_0} - 1) - \sigma_{coll})h_n \,.$$

Normal muscular tension $T_{m,n}$ is

$$T_{m,n} = \left(\frac{P_{a,n} + P_{v,n}}{2} - P_{ic,n}\right) r - T_{e,n} \,.$$

This gives $T_{max,n}$:

$$T_{max,n} = \frac{T_{m,n}}{\exp\left(-\left|\frac{r_n - r_m}{r_t - r_m}\right|^{n_m}\right)} \,.$$

μ_n, the normal value of μ, is zero so that:

$$T_{max0} = T_{max,n} \,.$$

Normal fractional arterial volume, $\mathrm{Vol_{art,n}}$ is calculated from AVRn via

$$\mathrm{Vol_{art,n}} = \mathrm{AVRn}/(1 + \mathrm{AVRn}) \,.$$

Baseline values of the NIRS quantities Hbt, HbO2 and HHb are given by:

$$\begin{aligned}
\mathrm{Hbt_n} &= \frac{1000}{4}\,[\mathrm{Hbtot_n}]\mathrm{Vol_{blood,n}} \\
\mathrm{HbO2_n} &= \frac{1000}{4}\,(\mathrm{Vol_{art,n}}[\mathrm{HbO_{2,a}}] + \mathrm{Vol_{ven}}[\mathrm{HbO_{2,v,n}}])\mathrm{Vol_{blood,n}} \\
\mathrm{HHb_n} &= \mathrm{Hbt_n} - \mathrm{HbO2_n}\,.
\end{aligned}$$

C.4 Oxygen transport and consumption

Directly set parameters

$\mathrm{CMRO_{2,n}} = 0.034$ mM s^{-1}. This value can be calculated from the value of 3.4 mL/100 g brain/min quoted by a number of authors, e.g. [4].

$[\mathrm{O}_{2,n}] = 0.024$ mM. Physiological mitochondrial O_2 concentrations of $20 - 30$ μM are quoted in [5]. [6] uses a value of 0.0262 mM for "intracellular oxygen", but the context is mitochondrial.

$\mathrm{Hbtot}, \mathrm{Hbtot_n} = 9.1$ mM. The value of 2.27 mM Hb used in the BRAINCIRC model equates to a blood haemoglobin concentration of about 154 g/L, consistent with the normal physiological range. Multiplying by 4 gives the number of O_2 binding sites.

Calculated parameters

The quantities ϕ and n_h in the relationship between dissolved oxygen and haemoglobin bound oxygen are calculated as follows: Traditionally the shape of saturation curves is represented by Hill equations of the form

$$\mathrm{SO2} = \frac{[\mathrm{O_2}]^{n_h}}{\phi^{n_h} + [\mathrm{O_2}]^{n_h}}$$

where SO2 is oxygen saturation. This solves to give

$$[\mathrm{O_2}] = \phi \left(\frac{\mathrm{SO2}}{1 - \mathrm{SO2}}\right)^{\frac{1}{n_h}} .$$

A value of $n_h = 2.5$ and half maximal oxygen partial pressure of 26 mmHg is found to fit the data well in [7]. Assuming the solubility of O_2 in blood is 0.0014 mM mmHg^{-1} gives $\phi = 0.036$ mM.

The quantity D_{O2} (in s^{-1}) is chosen to given normal oxygen supply at normal blood and mitochondrial oxygen concentrations, i.e.

$$D_{\mathrm{O2}} = \frac{J_{\mathrm{O2},n}}{([\mathrm{O}_{2,c,n}] - [\mathrm{O}_{2,n}])}\,. \tag{C.1}$$

$J_{O2,n}$ is set to $\mathrm{CMRO}_{2,\mathrm{n}}$, the normal value of CMRO_2. In order to calculate $[\mathrm{O}_{2,c,n}]$ we first need to calculate normal venous oxyhaemoglobin from normal arterial oxyhaemoglobin and delivery from the conservation equation

$$\mathrm{SvO2_n} = \mathrm{SaO2_n} - \frac{J_{\mathrm{O2},n}}{\mathrm{CBF_n}[\mathrm{Hbtot_n}]}\,.$$

Taking "typical" capillary haemoglobin to be

$$\mathrm{ScO2_n} = (\mathrm{SaO2_n} + \mathrm{SvO2_n})/2,$$

we can calculate typical capillary dissolved oxygen concentration from the dissociation curve

$$[\mathrm{O}_{2,c,n}] = \phi\left(\frac{\mathrm{ScO2_n}}{1 - \mathrm{ScO2_n}}\right)^{\frac{1}{n_h}}\,.$$

C.5 Mitochondria

Set parameters: general

$\mathrm{Vol_{mit}} = 0.067$, and $Z = 59.028$ mV are taken from [10]. It should be noted that the volume fraction of mitochondria is likely to show variation between tissues and individuals.

$p_{tot} = 20$ (or 12). This is the total number of protons pumped during the passage of four electrons from an initial reducing substrate to oxygen. The value of p_{tot} depends on which reducing substrate is used. Where the reducing substrate is NADH, p_{tot} is taken as 20 (i.e. 10 protons per NADH molecule). When the reducing substrate is succinate and the simplified model is being run, this number is decreased to 12 [9, 10].

$p_1 = 12$ (or 4), $p_2 + p_3 = 8$, following [9], and close to the values in [10]. [23] suggests that equal numbers of charges are transferred during the oxidative and reductive phases of the cytochrome-c-oxidase reaction, giving $p_2 = 4$ and $p_3 = 4$. When the reducing substrate is succinate p_1 becomes 4.

$\mathrm{cytox_{tis}} = 0.0055$ mmol (l tissue)$^{-1}$ [20]. Given the large changes in tissue cytochrome-c-oxidase content during development, it is possible that there is some physiological variation in this quantity, giving rise to quantitatively different ΔoxCCO signals in different individuals.

$\mathrm{CuA_{frac,n}} = 0.8$. The value of 0.82 is given for adult rat brain in [16]. The lower value of 0.673 for piglet is also quoted in [34]. In both cases there is a large standard deviation. The value of 0.8 is also consistent with the *in vitro* data for cytochrome c in [29], on the assumption that the redox states of cytochrome c and $\mathrm{Cu_A}$ are close. In any case, we expect the $\mathrm{Cu_A}$ centre *not* to be fully oxidised in normal circumstances.

$\Delta\Psi_n = 145$ mV consistent with values in [12].

$\Delta pH_n = pH_{m,n} - pH_o = 0.4$ [35]. $pH_o = 7$. This value of normal pH outside the mitochondria is used in [10] and the BRAINCIRC model. In the reduced model, pH_o can be seen as a control parameter. In the full model, various stimuli such as changes in PaCO2, or hypoxia, should be able to influence pH_o. Currently these pathways are omitted.

$dpH = 0.001$ and $C_{buffi} = 0.022$ M H^+/pH unit, are taken from [10].

$C_{im} = 6.7500 \times 10^{-3}$ mmol (l mito)$^{-1}$ mV^{-1} [8]. This parameter has an important influence on how a stimulus-induced change in Δp (e.g. via activation) translates into changes in ΔpH and $\Delta\Psi$.

Calculated parameters: general

$pH_{m,n} = pH_o + \Delta pH_n = 7.4$. Normal intramitochondrial pH is chosen to be 0.4 pH units greater than extramitochondrial pH. This is close to values obtained in simulations of [11] at normal parameter values and consistent with the ΔpH value in [35].

Normal PMF: $\Delta p_n = \Delta\Psi_n + Z\Delta pH_n$.

The total concentration of cytochrome-c-oxidase and hence Cu_A in mitochondria, $cytox_{tot}$ is set from the total concentration in tissue $cytox_{tis}$:

$$cytox_{tot} = cytox_{tis}/Vol_{mit}\,.$$

The normal oxidised fraction of Cu_A, i.e. $CuA_{frac,n}$ is used to calculate $CuA_{o,n}$ and $CuA_{r,n}$: via

$$CuA_{o,n} = CuA_{frac,n} cytox_{tot}, \quad CuA_{r,n} = cytox_{tot} - CuA_{o,n}\,.$$

Set parameters: reactions 1, 2 and 3

In this section $\mathcal{E}$ and $\mathcal{E}_0$ refer to reduction potentials and standard reduction potentials of half reactions respectively.

$\mathcal{E}_0(NADH) = -320$ mV from [12].

$N_t = 3$ mM. This value for the total mitochondrial NAD pool is taken from [10].

$N_{rat,n} = 9$. The normal NAD/NADH ratio is chosen to 9:1, i.e. mitochondrial NAD is assumed to be 10 percent reduced in normal circumstances. A wide range of values from about 5:1 to about 20:1 can be found in the literature, e.g. [17–19].Our value is within this range.

$D_{NADH} = 0.01$ mV. The extent to which demand activates glycolysis and the TCA cycle, and thus affects the NAD/NADH ratio, is obviously important. For example [32] suggests that an important effect of activation is glycolytic. The current value of D_{NADH} is chosen heuristically so that changes in demand have a small effect on the redox state of NADH. Increase in this parameter from 0.01 to 0.1 causes reduction of all modelled elements of the chain during functional activation.

$\mathcal{E}_0(UQ) = 60$ mV is the value for ubiquinone in [12].

$U_t = 1.35$ mM. This value for the total mitochondrial ubiquinone pool is taken from [10].

$\mathrm{U_{rat}} = 1$. This parameter is only needed for the simplified model and can be defined as the UQ/UQH_2, assumed to be determined by the experimental context.

$\mathrm{U_{rat,n}} = 1$. This parameter is only needed for the simplified model to calculate a normal equilibrium constant for reaction 1 and can be defined as the UQ/UQH_2 ratio such that at normal values of membrane potential and oxygen, we get flux f_n through the chain (in contexts where mitochondria are fed on succinate).

Simulations of [11] suggest that in normal circumstances the UQ/UQH_2 ratio is considerably less than the NAD/NADH ratio. When the value of supply (via parameter k_{DH} in that model) is adjusted so that NAD/NADH ratio becomes 9, the UQ/UQH_2 ratio is approximately 0.7 (at this value cytochrome c is about 85 percent oxidised, also consistent with our assumptions). It is not easy to derive from experimental work with succinate-fed mitochondria such as [29] accurate values of the UQ/UQH_2 ratio: However given that cytochrome c redox states in [29] are close to the $\mathrm{Cu_A}$ states observed *in vivo*, and hence presumably cytochrome c states *in vivo*, it is reasonable to assume that the UQ/UQH_2 ratio might also be similar. Further, simulations suggest that the model behaviour is insensitive to the precise value of $\mathrm{U_{rat}}$ and $\mathrm{U_{rat,n}}$ – a 10 percent change in either causes no more than a 1 percent change in baseline flux.

$\mathcal{E}_0(\mathrm{Cu_A}) = 247$ mV. This value is chosen to be somewhat less positive than the cyt c potential [13].

$\mathcal{E}_0(\mathrm{cyt\,a_3}) = 350$ mV, similar to the values in [14, 15]. The higher value in [10] is needed because in this model all proton pumping occurs prior to reduction of cyt $\mathrm{a_3}$. The cyt a3 redox potential is assumed to be independent of the reduction state of CuA, and vice versa, which is an oversimplification given the variety of redox interactions in the enzyme [15, 36].

$\mathrm{k_{3,0}} = 2.5 \times 10^5 \mathrm{mM^{-1}\,s^{-1}}$ is taken from [22]. As shown below this can be used to calculate a value of $\mathrm{a3_{frac,n}} \simeq 0.99$.

The seven parameters c_{k1}, c_{k2}, $c3$, $\Delta \mathrm{p}_{30}$, $L_{CV,0}$, r_{CV} and $\Delta \mathrm{p}_{CV0}$ between them control how the rates of reactions 1,2, and 3, and ATP synthase respond to changes in Δp . As they are specific to the structure of the model, they are not easily derivable from the literature. However they influence model behaviour during any process which affects Δp. The values chosen combine to give the behaviour described in the sections on functional activation and hypoxia.

$c_{k1} = 0.01$ is chosen heuristically. By setting this to be low, the effect of uncoupling is primarily on the reverse rate of electron transfer in reaction 1. It is possible that this parameter should change value depending on whether the full model or the simplified model is simulated.

$c_{k2} = 0.02$ is chosen heuristically, but again in a range where the effect of uncoupling is stronger on the reverse rate of electron transfer in reaction 2, following the simple model presented in [21].

$c3 = 0.11$. This parameter controls the maximum sensitivity of f_3 to changes in Δp and its value is set heuristically.

$\Delta p_{30} = \Delta p_n - 25$ mV. Simulations suggest that in order to get observed behaviour the rate f_3 should be sensitive to changes in Δp at normal membrane potentials. The point of maximum sensitivity is chosen to be somewhat lower than normal PMF.

Calculated parameters: reactions 1, 2 and 3

Normal reaction rates are set from $\mathrm{CMRO_{2,n}}$ by defining

$$f_{1,n} = f_{2,n} = f_{3,n} = f_n \equiv \mathrm{CMRO_{2,n}}/\mathrm{Vol_{mit}}.$$

The quantity E_1 is set in different ways depending on the situation we are trying to model. In general

$$E_1 = \mathcal{E}_0(\mathrm{Cu_A}) - \mathcal{E}(\mathrm{R})$$

where R is some reducing substrate. For the *in vivo* situation where the reducing substrate is primarily NADH, this becomes

$$E_1 = \mathcal{E}_0(\mathrm{Cu_A}) - \mathcal{E}_0(\mathrm{NADH}) + C_{\mathrm{NADH}}$$

where $C_{\mathrm{NADH}} = Z/2\log_{10}(\mathrm{NADH/NAD})$. Defining $\mathrm{N_{rat}} = \mathrm{NAD/NADH}$ with normal value $\mathrm{N_{rat,n}}$, we allow demand to influence the NAD redox state by setting

$$\mathrm{N_{rat}} = \frac{\mathrm{N_{rat,n}}}{u^{2D_{\mathrm{NADH}}}}.$$

The parameter D_{NADH} controls the sensitivity of the NAD redox state to changes in demand. From $\mathrm{N_t}, \mathrm{N_{rat,n}}$ and $\mathrm{N_{rat}}$ we get the NADH concentrations $\mathrm{NADH_n} = \mathrm{N_t}/(1+\mathrm{N_{rat,n}})$, and $\mathrm{NADH} = \mathrm{N_t}/(1+\mathrm{N_{rat}})$.

For readability, the full forms of E_1 for reducing substrates NADH and $\mathrm{UQH_2}$ are:

$$E_1(\mathrm{NADH}) = \mathcal{E}_0(\mathrm{Cu_A}) - \mathcal{E}_0(\mathrm{NADH}) + \frac{Z}{2}\log_{10}\left(\frac{\mathrm{N_{rat,n}}}{u^{2D_{\mathrm{NADH}}}}\right)$$

$$E_1(\mathrm{UQH_2}) = \mathcal{E}_0(\mathrm{Cu_A}) - \mathcal{E}_0(\mathrm{UQH_2}) + \frac{Z}{2}\log_{10}(\mathrm{U_{rat}}).$$

The normal value of E_1 is

$$E_{1,n} = \mathcal{E}_0(\mathrm{Cu_A}) - \mathcal{E}(\mathrm{R_n}),$$

where $\mathcal{E}(\mathrm{R_n})$ is the reduction potential at normal concentration of R. So when the reducing substrate is NADH,

$$E_{1,n} = \mathcal{E}_0(\mathrm{Cu_A}) - \mathcal{E}_0(\mathrm{NADH}) + \frac{Z}{2}\log_{10}(1/\mathrm{N_{rat,n}}),$$

and similarly for succinate as the reducing substrate

$$E_{1,n} = \mathcal{E}_0(\mathrm{Cu_A}) - \mathcal{E}_0(\mathrm{UQH_2}) + \frac{Z}{2}\log_{10}(1/\mathrm{U_{rat,n}})\,.$$

From the normal values of E_1 and Δp, it is possible to calculate a normal equilibrium constant for reaction 1, and hence normal value of $\mathrm{k_1}$:

$$\mathrm{Keq_{1,n}} = 10^{-1/Z(p_1\Delta \mathrm{p}_n/4 - E_{1,n})}, \quad \mathrm{k_{1,n}} = f_n/(\mathrm{CuA_{o,n}} - \mathrm{CuA_{r,n}}/\mathrm{Keq_{1,n}})\,.$$

When an *in vivo* situation is modelled with NADH as the main reducing substrate, we choose $\mathrm{k_{1,0}} = \mathrm{k_{1,n}NADH/NADH_n}$ where $\mathrm{k_{1,n}}$ is the value of k_1 at normal Δp and NADH levels. A similar methodology is applied when the reducing substrate is succinate.

E_2 is set as:

$$E_2 = \mathcal{E}_0(\mathrm{cyt\,a_3}) - \mathcal{E}_0(\mathrm{Cu_A})\,.$$

From the values of E_2 and normal value of Δp, it is possible to calculate a normal equilibrium constant for reaction 2, and hence normal value of $\mathrm{k_2}$:

$$\mathrm{Keq_{2,n}} = 10^{-1/Z(p_2\Delta \mathrm{p}_n/4 - E_2)}, \quad \mathrm{k_{2,n}} = f_n/(\mathrm{CuA_{r,n}a3_{o,n}} - \mathrm{CuA_{o,n}a3_{r,n}}/\mathrm{Keq_{2,n}})\,.$$

$\mathrm{k_3}$ is calculated from measured values of $\mathrm{k_{3,0}}$, i.e.

$$\mathrm{k_3} = \frac{\mathrm{k_{3,0}}(1+\exp(c3\,\Delta \mathrm{p_{30}}))}{\exp(c3\,\Delta \mathrm{p_{30}})}\,.$$

$\mathrm{a3_{r,n}}$ is calculated by inverting the definition of f_3 at normal values of all quantities:

$$\mathrm{a3_{r,n}} = \frac{f_n(1+\exp[-c3(\Delta \mathrm{p}_n - \Delta \mathrm{p_{30}})])}{\mathrm{k_3[O_{2,n}]}\exp[-c3(\Delta \mathrm{p}_n - \Delta \mathrm{p_{30}})]}\,.$$

This gives rise to a normal oxidised fraction of cyt $\mathrm{a_3}$ as $\mathrm{a3_{frac,n}} = (\mathrm{cytox_{tot}} - \mathrm{a3_{r,n}})/\mathrm{cytox_{tot}} \simeq 0.99$.

Set parameters: Complex V and proton leak

Three parameters determine the shape of response of the ATPase to changes in PMF: $L_{CV,0}$, $\Delta \mathrm{p}_{CV0}$ and r_{CV}. The functional form of L_{CV} is currently chosen to match qualitatively the function in [37]. As the function used in [37] is both complex and an approximation to a previous approximation in [35], rather than attempting to use it directly we chose instead a simple class of increasing functions which saturates at both large and small values of Δp. As mentioned above, the parameter values are chosen to be consistent with the behaviour described in the sections on functional activation and hypoxia, but are not necessarily the unique parameter values giving reasonable model behaviour.

$L_{CV,0} = 0.4$. This is the normal rate of ADP phosphorylation as a proportion of maximal phosphorylation rate – combined with Δp_{CV0} and r_{CV} it determines the maximum slope of the flux-Δp relationship for the ATPase. It is set so that at normal values Δp exerts some control over the rate of oxidative phosphorylation.

$\Delta p_{CV0} = 90$ mV and $r_{CV} = 5$. [35] ignores the possibility of reversibility of the enzyme (this effectively sets $r_{CV} \to \infty$). Here r_{CV} is given a finite value, following [37] (from which a value of $r_{CV} \approx 3$ can be inferred). The graphs in [37] can also be used to derive a somewhat higher value of Δp_{CV0} than used here. Investigating the effect of the shape of the flux-Δp relationship on the behaviour of the model is an important task for the future.

$L_{lk,frac} = 0.25$. [38] has the very high value of 0.85 for synaptosomes. The value chosen here lies in the range given for most tissues in [9]. However the effect of this parameter on model behaviour is clearly important for future exploration.

$k_{lk2} = 0.038$ mV^{-1}, taken from [10].

Calculated parameters: Complex V and proton leak

The normal value of L, the inward proton current through the membrane, is set to

$$L_n = p_{tot}\mathrm{CMRO}_{2,\mathrm{n}}/\mathrm{Vol}_{\mathrm{mit}},$$

since p_{tot} is precisely the number of protons pumped during reduction of one molecule of oxygen. A quantity $L_{lk,frac}$ is defined as the normal fraction of L passing through leak channels giving $L_{CV,frac} = 1 - L_{lk,frac}$ as the normal fraction passing through Complex V. This gives normal value of L_{lk} and L_{CV}:

$$L_{lk,n} = L_{lk,frac}L_n, \qquad L_{CV,n} = L_{CV,frac}L_n\,.$$

The constant L_{lk0} is calculated from normal proton motive force Δp_n and $L_{lk,n}$:

$$L_{lk0} = \frac{L_{lk,n}}{\exp(k_{lk2}\Delta p_n) - 1}\,.$$

The quantity k_{CV} is set by defining the control parameter $L_{CV,0}$ (see above):

$$L_{CV,0} \equiv \frac{1 - \exp(-k_{CV}(\Delta p_n - \Delta p_{CV0}))}{1 + r_{CV}\exp(-k_{CV}(\Delta p_n - \Delta p_{CV0}))}\,,$$

(normal u is 1 has been assumed) and inverting to give:

$$k_{CV} = -\frac{1}{\Delta p_n - \Delta p_{CV0}}\ln\left(\frac{1 - L_{CV,0}}{1 + r_{CV}L_{CV,0}}\right)\,.$$

The constant $L_{CV,max}$ is calculated as

$$L_{CV,max} = \frac{L_{CV,n}}{L_{CV,0}} \,.$$

D Calculating average capillary oxygen concentration

In order to check the validity of calculating average capillary oxygen concentration from the average of arterial and venous saturation, a simple distributed model was constructed and solved numerically. The model is constructed by dividing a capillary into N segments. Three species – oxyhaemoglobin, deoxyhaemoglobin and dissolved oxygen flow into and out of each segment. Dissolved oxygen diffuses out of the each segment and is consumed in mitochondria. A schematic representation is shown in Figure D.1.

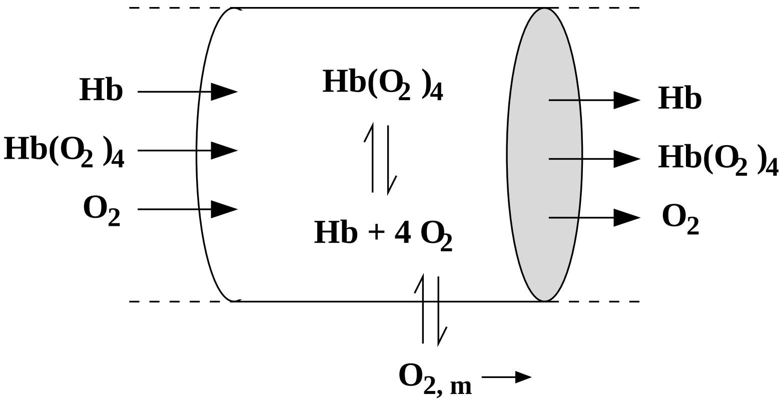

Fig. D.1. **The distributed model of a capillary.** Using the subscript j to represent concentrations in the jth segment, a given substance X (i.e. $Hb(O_2)_4$, Hb or O_2) flows into the jth segment at rate $q[X_{j-1}]$ and flows out at rate $q[X_j]$ where q is blood flow. The reaction $Hb + 4O_2 \rightleftharpoons Hb(O_2)_4$ takes place in segment j at rate $k_1[Hb_j][O_{2,j}]^{n_h} - k_2[Hb(O_2)_{4,j}]$. Oxygen is exchanged between capillary segment and mitochondria at rate $k_3([O_{2,j}] - [O_{2,m}])$ (where $O_{2,m}$ is mitochondrial oxygen), and mitochondrial oxygen is used up at rate $k_4[O_{2,m}]$. Details of the rate constants k_1, k_2, k_3 and k_4 are provided in the text.

Define $a_j \equiv [Hb_j]$, $b_j \equiv [Hb(O_2)_{4,j}]$, $c_j \equiv [O_{2,j}]$ for $j = 1, \ldots, N$, and let and $c_m \equiv [O_{2,m}]$. Further let a_0, b_0 and c_0 be arterial concentrations of Hb, $Hb(O_2)_4$, and O_2 respectively. Taking into account mitochondrial volume $\mathrm{Vol_{mit}}$ and segment volume $\mathrm{Vol_c}/N$ (where $\mathrm{Vol_c}$ is capillary volume) we get the following equations for the instantaneous change in concentrations:

$$\begin{aligned}
\dot{a_j} &= Nq(a_{j-1} - a_j)/\mathrm{Vol_c} - (k_1 a_j c_j^{n_h} - k_2 b_j) \quad j = 1, \ldots, N \\
\dot{b_j} &= Nq(b_{j-1} - b_j)/\mathrm{Vol_c} + (k_1 a_j c_j^{n_h} - k_2 b_j) \quad j = 1, \ldots, N \\
\dot{c_j} &= N[q(c_{j-1} - c_j) - k_3(c_j - c_m)]/\mathrm{Vol_c} - 4(k_1 a_j c_j^{n_h} - k_2 b_j) \quad j = 1, \ldots, N \\
\dot{c_m} &= -k_4 c_m + k_3 \frac{\left(\sum_{j=1}^{n} c_j - c_m\right)}{\mathrm{Vol_{mit}}} \,.
\end{aligned} \tag{D.1}$$

Parameters used in the distributed model are listed in Table D.1.

parameter	source	value and units
q	$\mathrm{CBF_n}$ in full model	0.01075 ml blood (ml brain tissue)$^{-1}$ s^{-1}
SaO2	full model	0.96
$\mathrm{Vol_c}$	$0.1\,\mathrm{Vol_{blood,n}}$	0.004 ml capillary blood (ml brain tissue)$^{-1}$
$\mathrm{Vol_{mit}}$	full model	0.067 ml mitochondria (ml brain tissue)$^{-1}$
ϕ	full model	0.036 mM
n_h	full model	2.5
$\mathrm{CMRO_{2,n}}$	full model	0.034 mM s^{-1}
$[\mathrm{O_{2,m}}]_n$	$[\mathrm{O}_{2,n}]$ in full model	0.024 mM
k_2	[see text]	1000 s^{-1}
k_1	$k_2\phi^{-n_h}$	4.067×10^6 mM$^{-n_h}$s^{-1}
k_3	[see text]	0.00948 s^{-1}
k_4	$\mathrm{CMRO_{2,n}}/[\mathrm{O_{2,m}}]_n/\mathrm{Vol_{mit}}$	22.355 s^{-1}
Hbt	one quarter of Hbtot in full model	2.275 mM
$\mathrm{b_0}$	SaO2 Hbt	2.184 mM
$\mathrm{a_0}$	$\mathrm{Hbt} - \mathrm{b_0}$	0.0091 mM
$\mathrm{c_0}$	$\phi[\mathrm{SaO2}/(1-\mathrm{SaO2})]^{1/n_h}$	0.1284 mM

Table D.1. The parameters used in the distributed model.

The model was calibrated so that normal flux was the same as that of the full model. q was set to be normal blood flow $\mathrm{CBF_n}$. The parameter k_4 was set so that at normal mitochondrial O_2 (the value used in the full model) we get normal $CMRO_2$ as used in the full model, i.e.

$$k_4 = \frac{\mathrm{CMRO_{2,n}}}{[\mathrm{O_{2,m}}]_n \mathrm{Vol_{mit}}} .$$

k_3 is then set to ensure normal mitochondrial O_2 at steady state, i.e. so that at steady state

$$\mathrm{Vol_{mit}} k_4 [\mathrm{O_{2,m}}]_n = k_3 \left(\sum_{j=1}^{N} c_j - [\mathrm{O_{2,m}}]_n \right) .$$

Since we have no *a priori* knowledge of the capillary distribution, this involves adjusting k_3 until the two sides of the equation are equal. The ratio of rates k_1 and k_2 is chosen to be ϕ^{-n_h} (where ϕ is the concentration of dissolved oxygen at half maximal saturation) to ensure that at equilibrium we get

the same dissociation curve used in the full model. k_2 is then set to be large ($k_2 = 1000$) to ensure rapid equilibriation of the dissociation reaction.

The model was solved at steady state for $N = 100$ to get the profiles of oxyhaemoglobin and dissolved oxygen along the segment. These profiles are plotted in Figure D.2. While the haemoglobin saturation profile is approximately linear, the concentration of dissolved oxygen is quite nonlinear, even with the model running at normal values of blood flow, arterial saturation and $CMRO_2$. This reflects the nonlinearity of the saturation function.

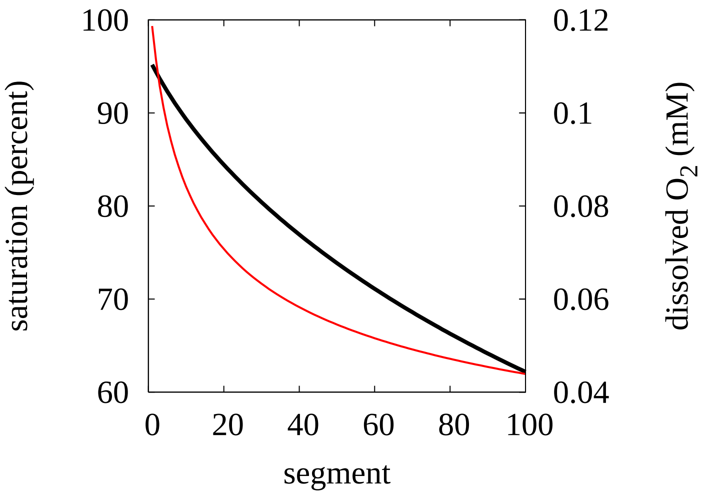

Fig. D.2. **Profiles of haemoglobin saturation and dissolved oxygen.** The profiles for haemoglobin saturation (black) and dissolved oxygen (red) along a capillary according to the distributed model with all parameters at normal values as listed in Table D.1.

In order to check the validity of calculating average capillary oxygen levels from the average of arterial and venous saturation, a "typical" capillary haemoglobin saturation of $\mathrm{ScO2} = (b_0 + b_{100})/(2\mathrm{Hbtot})$ was used to calculate an approximate capillary O_2 concentration of

$$\tilde{\mathrm{O}}_{2,\mathrm{c}} = \phi \left(\frac{\mathrm{ScO2}}{1 - \mathrm{ScO2}} \right)^{1/n_n} .$$

This approximate concentration was compared to the true average O_2 concentration

$$\mathrm{O}_{2,\mathrm{c}} = \frac{1}{100} \sum_{j=1}^{100} c_j$$

for a range of arterial O_2 saturations and values of blood flow. Some results from these comparisons are presented in Figure D.3.

Despite the nonlinear profile of oxygen concentration along a capillary, the errors introduced by choosing the approximate value of capillary oxygen concentration are surprisingly small, although they may become of concern during simulations of ischaemia.

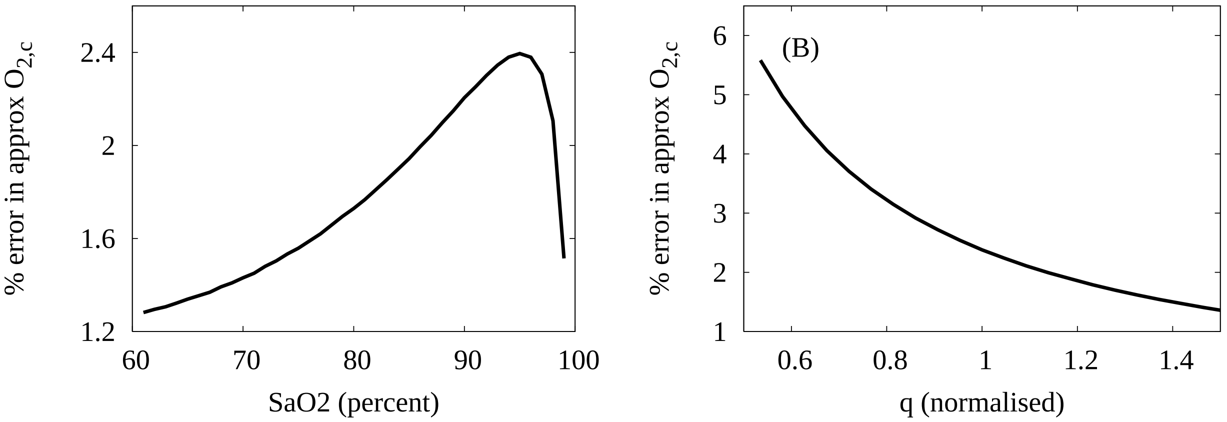

Fig. D.3. **Approximate arterial oxygen concentration compared to true average from the distributed model.** A) with q held at normal value, SaO2 was varied from 0.6 (60 percent) to 0.99 (99 percent). $100(\tilde{O}_{2,c}/O_{2,c} - 1)$ was calculated to give a percentage error in $\tilde{O}_{2,c}$. This error remained within 2.5 percent being greatest near the normal value of 96 percent saturation. B) With SaO2 held at normal value, q was varied from 50 percent of its normal value to 150 percent of its normal value. Again the percentage error in $\tilde{O}_{2,c}$ was calculated. $\tilde{O}_{2,c}$ remained within 6 percent of $O_{2,c}$ with maximum error occurring during ischaemia.

E A single compartment model and autoregulation curves

Despite the geometric complexity of the vascular system, it was found empirically that a model of the kind used in [1], but with a single resistive compartment was able to fit autoregulation data well. Associated with such a compartment are several parameters, in particular r_0, a typical radius associated with the passive response; r_m, a radius at which the active response is maximal; r_t and n_m, a radius and an exponent determining the shape of the active response; and h_0, vascular wall thickness when vessel radius is r_0. As vessel response is independent of the particular units chosen, r_0, r_m, r_t and h_0 can all be expressed in terms of a normal radius r_n, which is convenient to introduce. The autoregulatory response of the system depends on the above quantities, along with normal blood pressure $P_{a,n}$ and the autoregulatory sensitivity R_P which determines the effect of transmural pressure on smooth muscle response. Thus eight quantities, r_n, r_0, r_m, r_t, n_m, h_0, $P_{a,n}$ and R_P potentially determine the model autoregulation curve, although nondimensionalisation could reduce this to seven. Values chosen for different segments in [1] suggested that n_m, r_t and h_0 do not vary significantly along the tree. On the other hand r_m, r_0 and R_P had considerably different values along the tree, and simulations showed that the normal operating radius r_n was different for different segments. It was found that fixing r_m, r_t, n_m and h_0 at the values for the proximal arterial segment in [1], fixing r_n at an arbitrary value in the normal range, and varying $P_{a,n}$, R_P and r_0 sufficed to give good fits to published data sets. Two examples of best fits to data are presented in Figure E.1. In both cases the fitting algorithm was a simulated annealing process as described in [39], with the mean-square vertical distance between data set and autoregulation curve chosen as the objective function to be minimised.

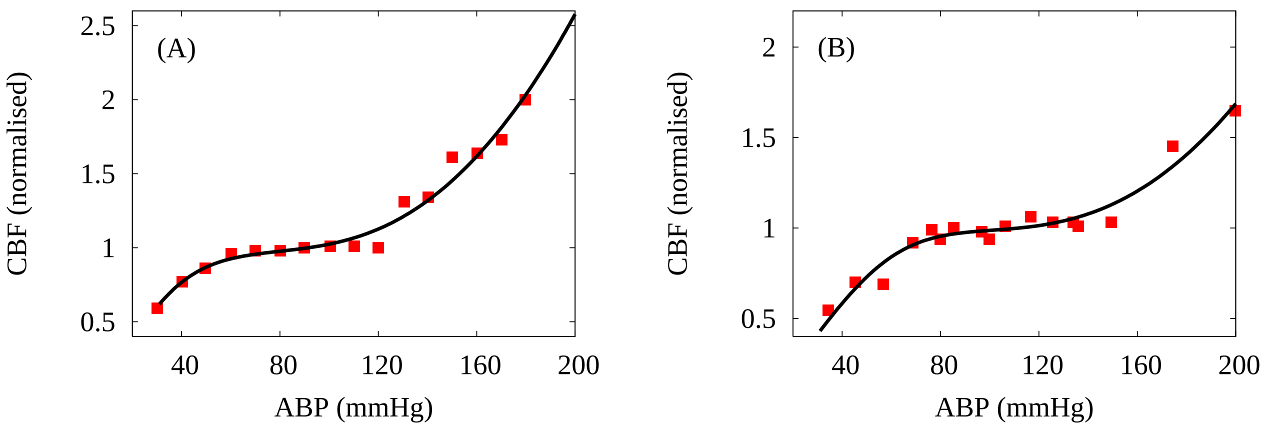

Fig. E.1. **The response of model steady state CBF to blood pressure changes.** A) Model autoregulation curve fitted to data from [26]. The following parameters were reset: $P_{a,n} = 91.6$, $R_P = 3.05$ and $r_0 = 0.015$. All other parameters were at their default values. B) Model autoregulation curve fitted to data from [25]. The best fit gave $P_{a,n} = 112.0$, $R_P = 3.28$ and $r_0 = 0.0133$.

It should be stressed that autoregulatory responses are liable to show considerable variability between individuals, and the values chosen should not be seen as absolute and unchanging. The actual values chosen give a curve in between the best fit curves for the above two data sets, and close to the curve produced by [1].

References

[1] Ursino M, Lodi CA (1998) Interaction among autoregulation, CO_2 reactivity, and intracranial pressure: a mathematical model. Am J Physiol Heart Circ Physiol 274:H1715–H1728.

[2] Banaji M, Tachtsidis I, Delpy D, Baigent S (2005) A physiological model of cerebral blood flow control. Math Biosci 194:125–173.

[3] Bazan NG, Braquet P, Ginsberg MD, editors (1992) Neurochemical correlates of cerebral ischaemia. Plenum Press, New York.

[4] Philips BJ, Armstrong IR, Pollock A, Lee A (1998) Cerebral blood flow and metabolism in patients with chronic liver disease undergoing orthotopic liver transplantation. Hepatology 27:369–376.

[5] Bellamy TC, Griffiths C, Garthwaite J (2002) Differential sensitivity of guanylyl cyclase and mitochondrial respiration to nitric oxide measured using clamped concentrations. J Biol Chem 277:31801–31807.

[6] Aubert A, Costalat R (2002) A model of the coupling between brain electrical activity, metabolism, and hemodynamics: Application to the interpretation of functional neuroimaging. NeuroImage 17:1162–1181.

[7] Keener J, Sneyd J (1998) Mathematical Physiology, volume 8 of *Interdisciplinary Applied Mathematics*. Springer, New York.

[8] Wu F, Yang F, Vinnakota KC, Beard DA (2007) Computer modeling of mitochondrial tricarboxylic acid cycle, oxidative phosphorylation, metabolite transport, and electrophysiology. J Biol Chem 282:24525–37.

[9] Brand MD (2005) The efficiency and plasticity of mitochondrial energy transduction. Biochem Soc Trans 33:897–904. doi:10.1042/BST20050897.

[10] Korzeniewski B, Zoladz JA (2001) A model of oxidative phosphorylation in mammalian skeletal muscle. Biophys Chem 92:17–34.

[11] Korzeniewski B (2001) Theoretical studies on the regulation of oxidative phosphorylation in intact tissues. Biochim Biophys Acta 1504:31–45.

[12] Nicholls DG, Ferguson SJ (2002) Bioenergetics 3. Academic Press.

[13] Moody AJ, Rich PR (1990) The effect of pH on redox titrations of haem a in cyanide-liganded cytochrome-c oxidase: experimental and modelling studies. Biochim Biophys Acta 1015:205–215.

[14] Dutton PL, Wilson DF, Lee C (1970) Oxidation-reduction potentials of cytochromes in mitochondria. Biochemistry 9:5077–5082.

[15] Nicholls P, Wrigglesworth J (1988) Routes of cytochrome a3 reduction. The neoclassical model revisited. Ann NY Acad Sci 550:59–67.

[16] Cooper CE, Delpy DT, Nemoto EM (1998) The relationship of oxygen delivery to absolute haemoglobin oxygenation and mitochondrial cytochrome oxidase redox state in the adult brain: a near-infrared spectroscopy study. Biochem J 332:627–632.

[17] Kimura K, Ukikusa M, Ozawa K, Tobe T (1978) Changes in mitochondrial redox state following an oral glucose load. Acta Diabetologica 15:283–286.

[18] Zhou L, Stanley WC, Saidel GM, Yu X, Cabrera ME (2005) Regulation of lactate production at the onset of ischaemia is independent of mitochondrial NADH/NAD+: insights from in silico studies. J Physiol 569:925–937.

[19] Rasmussen HN, van Hall G, Rasmussen UF (2002) Lactate dehydrogenase is not a mitochondrial enzyme in human and mouse vastus lateralis muscle. J Physiol 541:575–80.

[20] Brown GC, Crompton M, Wray S (1991) Cytochrome oxidase content of rat brain during development. Biochim Biophys Acta 1057:273–275.

[21] Petersen LC, Nicholls P, Degn H (1974) The effect of energization on the apparent Michaelis-Menten constant for oxygen in mitochondrial respiration. Biochem J 142:247–252.

[22] Chance B (1965) Reaction of Oxygen with the Respiratory Chain in Cells and Tissues. J Gen Physiol 49:163–188. doi:10.1085/jgp.49.1.163.

[23] Belevich I, Verkhovsky M, Wikström M (2006) Proton-coupled electron transfer drives the proton pump of cytochrome *c* oxidase. Nature 440:829–832.

[24] Reivich M (1964) Arterial P_{CO_2} and cerebral hemodynamics. Am J Physiol 206:25–35.

[25] Gao E, Young WL, Pile-Spellman J, Ornstein E, Ma Q (1998) Mathematical considerations for modelling cerebral blood flow autoregulation to systemic arterial pressure. Am J Physiol Heart Circ Physiol 274:H1023–H1031.

[26] Harper SL, Bohlen HG, Rubin MJ (1984) Arterial and microvascular contributions to cerebral cortical autoregulation in rats. Am J Physiol Heart Circ Physiol 246:H17–24.

[27] Villringer A, Chance B (1997) Non-invasive optical spectroscopy and imaging of human brain function. Trends in Neurosciences 20:435–442.

[28] Leung T, Elwell C, Henty J, Delpy D (2002) Simultaneous measurement of cerebral tissue oxygenation over the adult frontal and motor cortex during rest and functional activation. Adv Exp Med Biol 530:385–389.

[29] Wilson DF, Rumsey WL, Green TJ, Vanderkooi JM (1988) The oxygen dependence of mitochondrial oxidative phosphorylation measured by a new optical method for measuring oxygen concentration. J Biol Chem 263:2712–2718.

[30] Tachtsidis I, Jones H, Oliver C, Delpy DT, Smith M, et al. (2005) Investigation of the changes in

cerebral tissue oxygenation measured with near infrared spectroscopy in response to moderate hypercapnia. J Cereb Blood Flow Metab 25. doi:10.1038/sj.jcbfm.9591524.0193.
[31] Hoge R, Atkinson J, Gill B, Crelier G, Marrett S, et al. (1999) Linear coupling between cerebral blood flow and oxygen and consumption in actiated human cortex. PNAS 96:9403–9408.
[32] Mintun MA, Lundstrom BN, Snyder AZ, Vlassenko AG, Schulman GL, et al. (2001) Blood flow and oxygen delivery to human brain during functional activity: Theoretical modeling and experimental data. Proc Natl Acad Sci USA 98:6859–64.
[33] Edvinsson L, Krause DN, editors (2002) Cerebral Blood Flow and Metabolism. Lippincott Williams & Wilkins.
[34] Springett R, Newman J, Cope M, Delpy DT (2000) Oxygen dependency and precision of cytochrome oxidase signal from full spectral NIRS of the piglet brain. Am J Physiol Heart Circ Physiol 279:H2202–2209.
[35] Magnus G, Keizer J (1997) Minimal model of β-cell mitochondrial Ca^{2+} handling. Am J Physiol Cell Physiol 273:C717–C733.
[36] Blair D, Ellis J WR, Wang H, Gray H, Chan S (1986) Spectroelectrochemical study of cytochrome c oxidase: pH and temperature dependences of the cytochrome potentials. Characterization of site-site interactions. J Biol Chem 261:11524–11537.
[37] Cortassa S, Aon MA, Marbán E, Winslow RL, O'Rourke B (2003) An integrated model of cardiac mitochondrial energy metabolism and calcium dynamics. Biophys J 84:2734–2755.
[38] Joyce OJP, Farmer MK, Tipton KF, Porter RK (2003) Oxidative phosphorylation by in situ synaptosomal mitochondria from whole brain of young and old rats. J Neurochem 86:1032–1041.
[39] Press WH, Teukolsky SA, Vetterling WT, Flannery BP (1992) Numerical Recipes in C. Cambridge University Press.
